# Comparative genomics and pangenome-oriented studies reveal high homogeneity of the agronomically relevant enterobacterial plant pathogen *Dickeya solani*

**DOI:** 10.1186/s12864-020-06863-w

**Published:** 2020-06-29

**Authors:** Agata Motyka-Pomagruk, Sabina Zoledowska, Agnieszka Emilia Misztak, Wojciech Sledz, Alessio Mengoni, Ewa Lojkowska

**Affiliations:** 1 Laboratory of Plant Protection and Biotechnology, Intercollegiate Faculty of Biotechnology University of Gdansk and Medical University of Gdansk, 58 Abrahama Street, 80-307 Gdansk, Poland; 2 Present address: Institute of Biotechnology and Molecular Medicine, 3 Trzy Lipy Street, 80-172 Gdansk, Poland; 3 Department of Biology, University of Florence, 6 Madonna del Piano Street, 50019 Sesto Fiorentino, Italy

**Keywords:** Soft rot, Blackleg, Pectinolytic bacteria, *Erwinia chrysanthemi*, *Pectobacteriaceae*, Next-generation sequencing, Whole genome sequencing, Pacific biosciences, Clusters of orthologous groups, Average nucleotide identity

## Abstract

**Background:**

*Dickeya solani* is an important plant pathogenic bacterium causing severe losses in European potato production. This species draws a lot of attention due to its remarkable virulence, great devastating potential and easier spread in contrast to other *Dickeya* spp. In view of a high need for extensive studies on economically important soft rot *Pectobacteriaceae*, we performed a comparative genomics analysis on *D. solani* strains to search for genetic foundations that would explain the differences in the observed virulence levels within the *D. solani* population.

**Results:**

High quality assemblies of 8 de novo sequenced *D. solani* genomes have been obtained. Whole-sequence comparison, ANIb, ANIm, Tetra and pangenome-oriented analyses performed on these genomes and the sequences of 14 additional strains revealed an exceptionally high level of homogeneity among the studied genetic material of *D. solani* strains. With the use of 22 genomes, the pangenome of *D. solani*, comprising 84.7% core, 7.2% accessory and 8.1% unique genes, has been almost completely determined, suggesting the presence of a nearly closed pangenome structure. Attribution of the genes included in the *D. solani* pangenome fractions to functional COG categories showed that higher percentages of accessory and unique pangenome parts in contrast to the core section are encountered in phage/mobile elements- and transcription- associated groups with the genome of RNS 05.1.2A strain having the most significant impact. Also, the first *D. solani* large-scale genome-wide phylogeny computed on concatenated core gene alignments is herein reported.

**Conclusions:**

The almost closed status of *D. solani* pangenome achieved in this work points to the fact that the unique gene pool of this species should no longer expand. Such a feature is characteristic of taxa whose representatives either occupy isolated ecological niches or lack efficient mechanisms for gene exchange and recombination, which seems rational concerning a strictly pathogenic species with clonal population structure. Finally, no obvious correlations between the geographical origin of *D. solani* strains and their phylogeny were found, which might reflect the specificity of the international seed potato market.

## Background

*Dickeya* spp. together with *Pectobacterium* spp. belong to the family *Pectobacteriaceae* [1] and are causative agents of economically important soft rot and blackleg diseases affecting various crops, vegetables and ornamentals worldwide [2]. These bacterial phytopathogens decay host tissue due to the production of a broad range of plant cell wall degrading enzymes (PCWDEs) i.e. pectinases (pectate and pectin lyases, polygalacturonases, pectin-methyl and acetyl esterases), cellulases and proteases, which are secreted via type I or II secretion systems [3, 4]. Because of the activities of PCWDEs, these necrotrophic bacteria get access to valuable sources of nutrients accumulated within the plant cell. Other worth mentioning virulence factors of *Pectobacteriaceae* include biofilm formation [5], motility [6], siderophores production [7], lipopolysaccharide [8] or synthesis of bacteriocins [7]. Such a molecular or adaptive repertoire takes part in progression of the incited infection. However, three crucial requirements need to be fulfilled for the development of disease symptoms: the pathogen should be virulent, the plant host susceptible and the encountered environmental conditions favourable for disease progression [9]. Typical blackleg symptoms comprise water-soaked, blackened stem base in addition to chlorosis and wilting of the leaves [2]. Often the progeny tubers do not develop and in the most severe cases there is a noticeable lack of emergent plants [2]. Regarding soft rot, slimy, water-soaked maceration areas are observable in the inner parenchymatous plant tissue. These zones, if exposed to air, turn brown or black with release of watery exudates [2, 10]. Assessment of the total economic impact of these diseases is demanding as *Pectobacterium* and *Dickeya* spp. are present on various plant hosts in diverse geographical regions where miscellaneous seed certification policies remain in force [11].

The pectinolytic bacterial species, which is in focus of this work, belongs to the genus *Dickeya*. The *Dickeya* genus was established in 2005 [12] to comprise several former members of at first *Erwinia* [13] and subsequently *Pectobacterium* [14] genera. To date, ten species i.e. *Dickeya aquatica* [15], *Dickeya chrysanthemi* [12], *Dickeya dadantii* (including *D. dadantii* subsp. *dadantii* and *D. dadantii* subsp. *dieffenbachiae* [12, 16]), *Dickeya dianthicola* [12], *Dickeya fangzhongdai* [17], *Dickeya lacustris* [18], *Dickeya paradisiaca* [12], *Dickeya solani* [19], *D. undicola* [20] and *Dickeya zeae* [12] are classified to the *Dickeya* genus. It is worth noting that *D. solani* has drawn a lot of attention ever since its first appearance in Europe in 2004–2005 [19, 21–23]. Outgrouping of uniform isolates belonging to the *Dickeya* genus was spotted independently, basing on the sequences of 16S rRNA [24], *recA* [25, 26] or *dnaX* genes [19, 23], in addition to Repetitive Extragenic Palindromic-PCR (REP-PCR) profiling [23]. Further support for homogeneity of these isolates was provided by whole-cell Matrix-Assisted Laser Desorption Ionization Time-Of-Flight Mass Spectrometry (MALDI-TOF MS), Pulse Field Gel Electrophoresis (PFGE) of total genomic DNA cut with *Xba*I or I-*Ceu*I restriction enzymes, PCR-based fingerprinting with Enterobacterial Repetitive Intergenic Consensus (ERIC) and BOX primers, comparison of the sequences of intergenic spacer (IGS) in addition to broadening the pool of the analysed housekeeping genes by including *dnaN*, *fusA*, *gapA*, *gyrA*, *purA*, *rplB* and *rpoS* sequences [19, 27–29]. Even though the observed relatedness in DNA-DNA hybridization (DDH) experiments between the type strains of *D. solani* and *D. dadantii* equalled 72%, therefore exceeding the cut-off threshold for species delineation [30], the performed pairwise Average Nucleotide Identity (ANI) calculation with 0.94 value gave contradictory results in favour of separation of these two taxa [19].

Official establishment of *D. solani* as a distinct clonal species dates back to 2014 [19]. Since then major scientific efforts have been made to provide insight into the occurrence, epidemiology, detection methods, taxonomic position, metabolic profiles, regulation of transcription, genetics and genomics of this phytopathogen [19, 27, 28, 31–39]. The presence of *D. solani* strains was reported in Europe and beyond, e.g. in the Netherlands [19], Belgium [40], Israel [35], Turkey [41], Finland [28], Norway [42], Portugal [31], Czech Republic [43], Denmark [43], United Kingdom [44], Northern Ireland [45], Greece [46], France [47], Switzerland [48], Spain [49], Slovenia [50], Georgia [51], Russia [52], Germany [32], Brazil [53] and China [54]. Notably, the tested isolates originated from a limited number of plants including potato [27, 28, 35], hyacinth [23] and iris [19], which might be related to previous assumptions on strict linkage between highly specialized pathogens of clonal origin and their host [19, 55]. Remarkable virulence, great devastating potential and easier spread of *D. solani* strains in contrast to other *Dickeya* spp. was observed by several research groups [21, 27, 28, 56, 57]. Therefore, there were attempts undertaken to explain foundations of these phenomena on the levels of genomes, transcriptomes and metabolomes [31–33, 38, 39, 47, 58, 59]. It is worth noting though that the majority of genome-oriented research conducted so far benefited from a limited number of whole genome sequences (WGS) [31, 38, 47, 58, 60, 61], impeding broad insight into the intraspecies variation of *D. solani*. A pangenome-related study is a potent strategy to address comprehensive description of genomic diversity within a bacterial species and to suggest possible genetic determinants for the noted phenotypic differences [31, 62, 63]. ‘Pangenome’ covers all genes detected in a certain bacterial species, while ‘core genome’ comprises the genes present in all the analysed strains, ‘dispensable genome’ encloses the genes observed in two or more strains and ‘unique genome’ consists of the genes detected just in a single bacterial isolate [64]. Undertaking pangenome-based approach allows to state the amount of whole sequenced genomes that would satisfactorily reflect the genetic repertoire of a studied species [31, 63, 65]. If such a number of WGS is reached, the pangenome might be described as closed.

In this study, we aimed at exploiting comparative genomics and pangenome-oriented tools for providing closer insight into biodiversity within the *D. solani* species. For this purpose, 8 de novo sequenced, assembled and annotated WGS of *D. solani* strains of diverse origin and year of isolation were acquired. The utilized analytic tools provided insight into extraordinarily high homogeneity among the available 22 *D. solani* genomes. Importantly, such a number of sequences turned out to be sufficient to report in this work an almost closed status of the pangenome of *D. solani* species.

## Results

### *D. solani* genomic assemblies

The newly sequenced genomes of 8 *D. solani* strains (Table 1) were assembled into 1–7 scaffolds with no N bases (Table 2) from the PacBio reads with the use of the genome assembly pipeline that we previously described [31]. This method profits solely from PacBio RSII raw reads that are at first filtered from adapters with the use of SMRT Analysis v. 2.3 (Pacific Biosciences, USA) and then corrected, trimmed and assembled with the use of Canu v. 1.5 [66]. Getting consensus and variant calling was achieved with Quiver (SMRT Analysis v. 2.3) [67] and final functional annotation was conducted with Prokka v. 1.12 [68]. The size of these genomes ranged from 4,882,124 bp to 4,934,537 bp, in the case of IFB0487 and IFB0421 *D. solani* strains, respectively (Table 2). The largest contig of the acquired assemblies varied in size from 4,934,537 bp to 2,394,283 bp regarding either IFB0421 or IFB0311 (Table 2). N_50_, which refers to the minimum length of contigs in which half of the bases of the assembly are covered, ranged from 755,734 bp to 4,934,537 bp (for IFB0695 or IFB0421; Table 2). L_50_, describing the number of contigs that comprise half of the genome size, spanned from 1 to 2 (Table 2). The calculated GC content falls within the range of 56.23 to 56.25 (Table 2). None of the contigs from de novo assembled *D. solani* genomes has been assigned to the sequences of plasmid origin as computed with the use of PlasmidFinder [69]. According to Prokka-based [68] annotation, the newly sequenced genomes of *D. solani* strains contained in total from 4304 to 4608 genes (in the case of IFB0212 and IFB0417, respectively; Table 1). The number of protein-coding genes varied from 4143 (IFB0212) to 4446 (IFB0417), while the quantities of the annotated rRNA and tRNA amounted to 18–22 and 72–75, respectively (Table 1).

**Table 1:** *Dickeya solani* strains subjected to de novo sequencing in the frames of this study in addition to their genomic contents

Genome nos /strain nos	Strain			Genome				
				Total number of genes	Number of genes encoding			
	Country, region^a^	Host, year of isolation	Literature reference		Proteins	rRNA	tRNA	tmRNAs
IFB0167	Poland, Lower Silesian Voivodeship	Potato cv. Fresco, 2009	[27]	4308	4146	22	75	1
IFB0212	Poland, Mazovian Voivodeship	Potato, 2010	[29]	4304	4143	18	72	1
IFB0231 (VIC-BL-25)	Finland, Liminka	Potato cv. Victoria, 2008	[28]	4313	4151	22	75	1
IFB0311	Poland, Pomeranian Voivodeship	Potato cv. Innovator, 2011	[27]	4306	4144	20	74	1
IFB0417	Portugal, Santarem	Potato cv. Lady Rosetta, 2012	This study	4608	4446	22	75	1
IFB0421	Portugal, Santarem	Potato cv. Lady Rosetta 2012	This study	4349	4187	22	75	1
IFB0487	Poland, Podkarpackie Voivodeship	Potato cv. Vineta, 2013	[27]	4572	4409	22	75	1
IFB0695	Poland, Kuyavian-Pomeranian	Potato cv. Arielle, 2014	This study	4337	4172	22	75	1

^a^The geographical locations of the isolated strains: IFB0167 - Wawrzyszow 50°73′12″ N 17°23′58″ E, IFB0212 - Mlochow 52°02′35.76″ N 20°46′4.01″ E, IFB0231 - High Grade seed potato growing region 64°48′35.46″ N 25°24′55.62″ E, IFB0311 - Lebork 54°32′11.181″ N 17°44′56.144″ E, IFB0417 and IFB0421 39°12′0″ N 8°42′0″ W, IFB0487 - Zdziechowice 50°47′00″ N 22°07′00″ E, IFB0695 - Niwy 53°34′39.443″ N 17°25′49.649″ E. For the origin and the annotated genomic features of the herein included *Dickeya solani* reference strains see our former study Golanowska et al. (2018) [31]

**Table 2:** Basic statistics in addition to the assembly quality metrics for the studied *D. solani* genomes

Genome	No. of scaffolds	No. of N bases	Genome size (bp)	Largest contig (bp)	N_50_	L_50_	%GC	Genbank accession no.	Reference
**IFB0167**	**1**	**0**	**4,922,289**	**4,922,289**	**4,922,289**	**1**	**56.25**	**CP051457**	**This study**
**IFB0212**	**2**	**0**	**4,909,935**	**3,946,010**	**3,946,010**	**1**	**56.25**	**JABAON000000000**	**This study**
**IFB0231**	**1**	**0**	**4,924,702**	**4,924,702**	**4,924,702**	**1**	**56.24**	**CP051458**	**This study**
**IFB0311**	**3**	**0**	**4,913,261**	**2,394,283**	**1,850,246**	**2**	**56.24**	**JABAOO000000000**	**This study**
**IFB0417**	**1**	**0**	**4,924,102**	**4,924,102**	**4,924,102**	**1**	**56.24**	**CP051459**	**This study**
**IFB0421**	**1**	**0**	**4,934,537**	**4,934,537**	**4,934,537**	**1**	**56.24**	**CP051460**	**This study**
**IFB0487**	**4**	**0**	**4,882,124**	**3,440,832**	**3,440,832**	**1**	**56.23**	**JABAOP000000000**	**This study**
**IFB0695**	**7**	**0**	**4,904,769**	**2,442,930**	**755,734**	**2**	**56.25**	**JABAOQ000000000**	**This study**
IFB0099	1	0	4,932,920	4,932,920	4,932,920	1	56.24	CP024711	[31, 76]
IFB0158	37	395	4,879,070	772,123	360,663	5	56.24	PENA00000000	[31]
IFB0221	38	394	4,878,255	774,432	360,663	5	56.24	PEMZ00000000	[31]
IFB0223	1	0	4,937,554	4,937,554	4,937,554	1	56.24	CP024710	[31]
IPO 2222	1	9200	4,867,258	4,867,258	4,867,258	1	56.22	AONU01000000	[44]
GBBC 2040	1	27,548	4,860,047	4,860,047	4,860,047	1	56.34	AONX01000000	[44]
MK10	3	3800	4,935,237	4,934,019	4,934,019	1	56.21	AOOP01000000	[44]
MK16	3	2100	4,870,382	4,865,372	4,865,372	1	56.23	AOOQ01000000	[44]
D s0432–1	4	0	4,904,518	2,278,175	1,562,114	2	56.20	AMWE01000000	[38]
PPO 9019	24	30	4,866,823	1,553,733	485,395	3	56.25	JWLS01000000	[39]
PPO 9134	22	187	4,870,830	1,553,748	485,873	3	56.24	JWLT01000000	[39]
RNS 05.1.2A	37	0	4,985,571	570,255	305,078	7	56.13	JWMJ01000000	[39]
RNS 07.7.3B	24	325	4,871,815	688,619	485,311	4	56.24	JWLR01000000	[39]
RNS 08.23.3.1A	1	12,124	4,923,743	4,923,743	4,923,743	1	56.25	AMYI01000000	[60]

The genomes depicted in bold have been de novo sequenced and assembled in the frames of this research. The versions of the included reference genomes are the ones downloaded from the Genbank database for Golanowska et al. 2018 [31]

Genomic contents and assembly statistics for the herein reported newly-sequenced *D. solani* genomes have been juxtaposed to these attributed to 14 reference *D. solani* sequences (versions of the genomes available in the Genbank database at a time of conducting research have been included; Table 2). The numbers of scaffolds building up the utilized reference genomes are considerably higher (1–38) than the quantities of scaffolds present in 8 de novo sequenced ones (4 are closed, while the remaining ones consist of 2–7 scaffolds; Table 2). Also, the vast majority of reference genomic sequences contain N bases, reaching even the number of 27,548 (GBBC 2040). Other quality metrics of reference assemblies like the largest contig (> 570,255 bp), N_50_ (> 305,078 bp) or L_50_ (< 7) are also in favour of the genome assembly pipeline used for the newly sequenced genomes. Moreover, it is worth noting a significantly higher variation (56.13–56.34) in the %GC among the reference genomes than de novo sequenced ones (Table 2).

Interestingly, the stated quantities of tRNA (Table 1) were often lower in the reference genomes, even though the range from 60 to 75 was broader [31]. Regarding rRNA, solely 1 to 4 such genes were annotated for the included versions of the reference genomes of PPO 9019, RNS 05.1.2A, RNS 07.7.3B, IPO 2222, GBBC 2040, MK10, MK16, PPO 9134, IFB0158 and IFB0221 strains [31], in contrast to 18–22 detected in the herein reported de novo sequenced genomes (Table 1).

Taking into consideration that genes coding for 5S, 16S and 23S rRNAs are typically organized into operons encountered in multiple copies, i.e. 1–14 [70], within the bacterial chromosome, such a low number of annotated rRNAs disagrees with the current biological knowledge. Thus, we postulate that the number of the annotated rRNA-encoding genes might be regarded as an informative marker of the achieved quality of de novo assembly of *D. solani* genomes in view of the fact that highly similar sequences of rRNAs were previously reported to potentially disrupt, due to the occurrence of both highly conserved and variable regions, the assembling process that is typically based on de Bruijn graphs [71]. It should be noted that the genomes possessing a low number of rRNA-coding genes have been assembled from the data generated by Illumina or 454 pyrosequencing platforms with the use of assemblers handling short length reads [31]. For example, the IPO 2222 genome available currently (13.02.20) in the GenBank database was reassembled from both PacBio and Illumina reads and harbours 22 rRNA-encoding genes in contrast to the number of three annotated for the here discussed version [31].

### Structural similarities between *D. solani* genomes

**Fig. 1:**
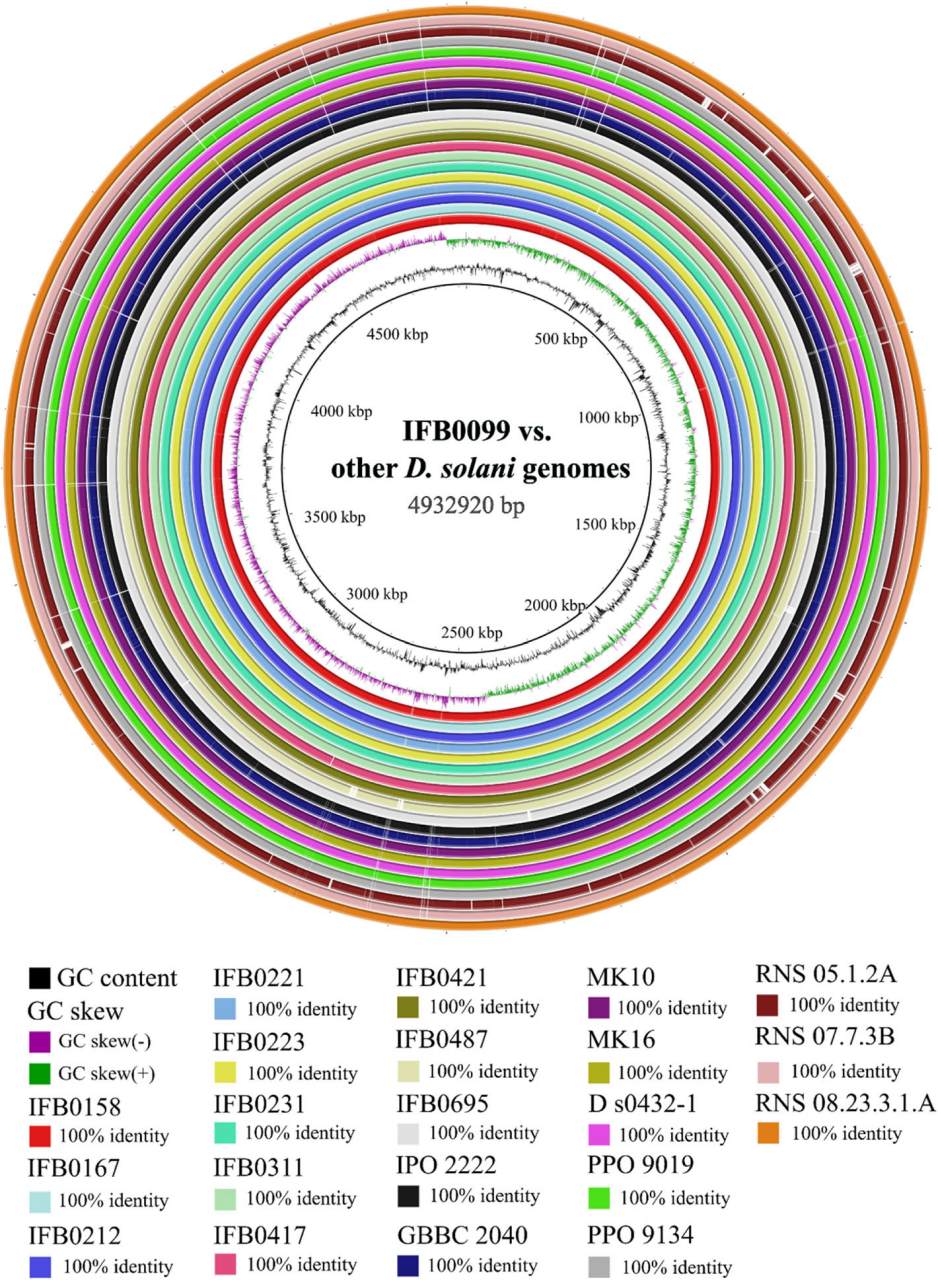
Whole genome comparison for 22 *Dickeya solani* strains. BLAST Ring Image Generator [72] software was implemented. *D. solani* IFB0099 was used as a reference. Two first rings correspond to the GC content and GC skew, respectively. Each of the depicted rings refers to one *D. solani* genome according to the listed coloration. White regions mark dissimilarities. The identities are based on BLAST calculations

Large scale BLAST comparison of de novo sequenced and reference *D. solani* genomes, computed with the use of BLAST Ring Image Generator (BRIG) [72], revealed an exceptionally high level of homogeneity among the studied 22 genomes (Fig. 1). The de novo sequenced genomes of *D. solani*: IFB0167, IFB0212, IFB0231, IFB0311, IFB0417, IFB0421, IFB0487, IFB0695 (Table 1), in addition to IFB0158, IFB0221, IFB0223, RNS 08.23.3.1A and D s0432–1, possess a nearly identical genomic structure to that of IFB0099 (Fig. 1), regardless of the sequencing method used or the closed/draft status of the genome assembly. A notable absence of certain genomic regions is a repeating feature in the case of other *D. solani* genomes, namely IPO 2222, GBBC 2040, MK10, MK16, PPO 9019 and PPO 9134 (Fig. 1). Some but not all of these sites are likewise not present in the genome of RNS 07.7.3B (Fig. 1). Undoubtedly, the genome of RNS 05.1.2A stands out from the pool of the tested sequences, not only taking into consideration the number, but also the size of the missing regions. It is also worth considering that the genomes of IFB0487 and IFB0695 lack quite large parts of DNA sequences present in the reference IFB0099 genome (Fig. 1). Putatively, it might be associated with the draft character of these genomic assemblies as the number of contigs is reflected in the number of computed synteny blocks. However, the presence of polymorphic sites in these regions cannot be excluded for sure due to the fact that in many cases incompleteness of a bacterial genomic assembly tends to result from the occurrence of repetitive sequences [73].

Basing genome comparisons on ANI values allows to avoid the bias linked with sequence selections and errors [74]. As this way of genomic distance determination takes advantage of whole-sequence information at high resolution of single nucleotides, three methods of pairwise genome comparisons, i.e. BLAST+ calculation of ANI (ANIb), MUMmer calculation of ANI (ANIm) and computation of the correlation indexes of the tetra-nucleotide signatures (Tetra) were utilized for proving an extraordinarily high similarity level between the analysed 22 *D. solani* genomes.

In more detail, the vast majority of ANIb values exceeded 99.96, reaching even 100.00 for over a dozen of juxtapositions (Supplementary Table 1). Similarly, in the case of ANIm, 99.98 was often reached, though no 100.00 values were acquired (Supplementary Table 2). It is also worth noticing that a high percentage of all the compared *D. solani* genomes have been successfully aligned (91.57–99.79 for ANIb and 93.26–100.00 for ANIm; Supplementary Tables 1 and 2). In addition, 1.0 correlation of the tetra-nucleotide signatures was likewise not rarely exhibited by the studied sequences (Supplementary Table 3). Regarding the observed differences, the genome of RNS 05.1.2A strain diverged to the greatest extent from the other sequences studied (Supplementary Tables 1, 2 and 3). ANIb values acquired for comparisons including this genome ranged from 98.55 (vs. PPO 9019) to 98.68 (vs. either RNS 07.7.3B or RNS 08.23.3.1A) (Supplementary Table 1), ANIm varied from 98.71 (towards PPO 9019) to 98.82 (in contrast to RNS 07.7.3B) (Supplementary Table 2), while tetra nucleotide correlation coefficients differed from 0.99976 (vs. either IFB0417 or IFB0487) to 0.99987 (in comparison to MK16) (Supplementary Table 3). ANIb (98.55–99.93) and ANIm (98.71–99.92) calculations also pointed to PPO 9019 and PPO 9134 as the genomes slightly standing out from the others tested (Supplementary Tables 1 and 2), though this deviation was not supported by the correlation coefficients-based method (Supplementary Table 3).

### Further insight into the pangenome composition of *D. solani*

**Fig. 2:**
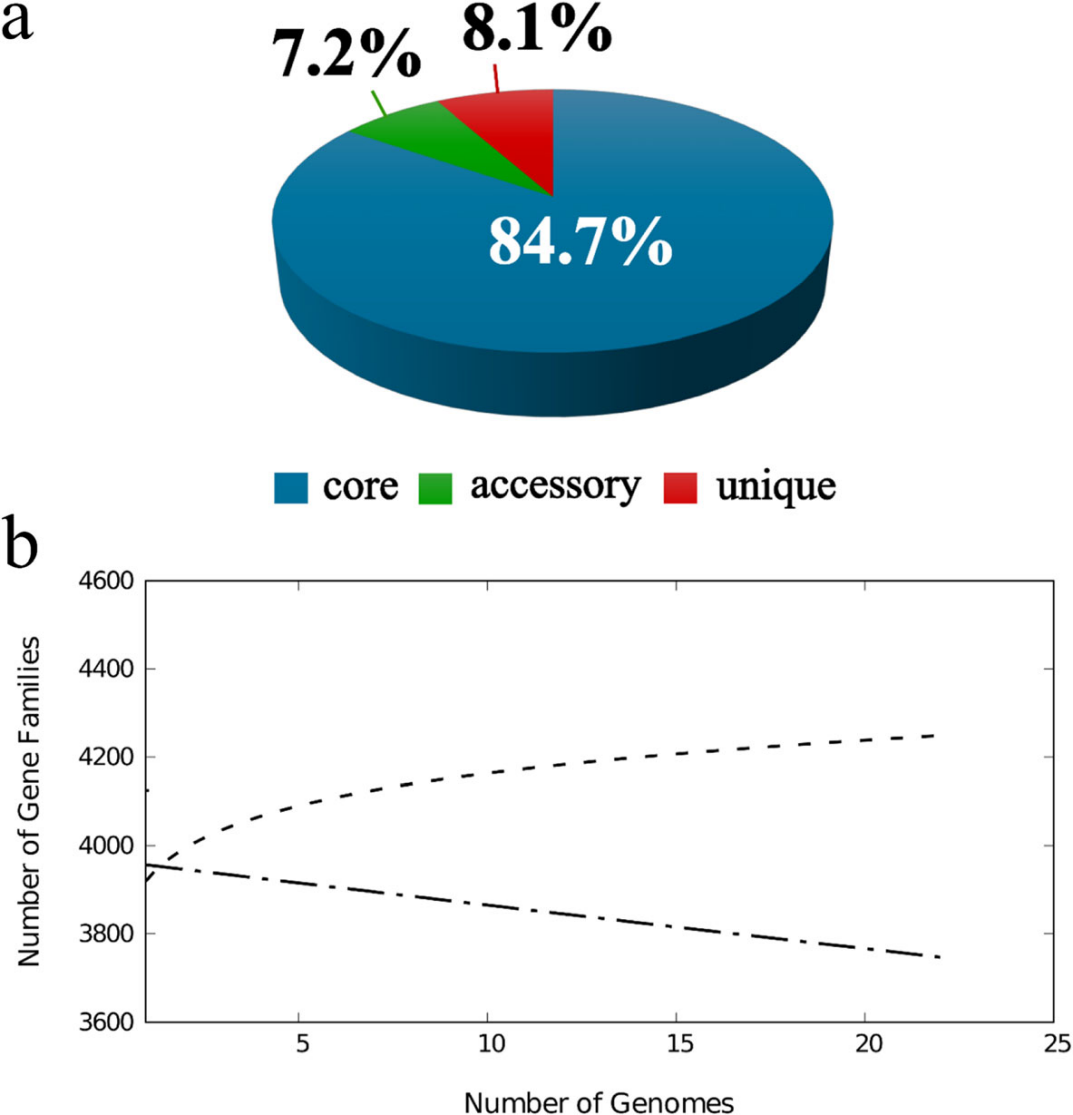
The pangenome profile of *Dickeya solani* species. BPGA [75] was implemented for the calculations. Abundancy of the core, accessory and unique pangenome fractions within the pangenome of *D. solani* (**a**). Total number of distinct gene families referring to the pangenome size (dashed line; power-fit curve equation: f(x) = 3924.52 ∙ x^0.0256574^) in addition to the number of core gene families (dash-dotted line; exponential curve equation: f1(x) = 3966.10 ∙ e^-0.00258611x^) are plotted against the number of genomes included (**b**)

The first glimpse into the structure of *D. solani* pangenome was provided in our former study [31]. In that work, Mauve-based calculation on 14 (5 closed and 9 draft) *D. solani* genomes showed that 74.8% (3756 genes) of the gene pool grouped into the core, 11.5% (574 genes) to the accessory and 13.7% (690 genes) to the unique pangenome fraction. In the current research, we significantly enlarged the number of the included *D. solani* genomes to 22 and applied another software named Bacterial Pan Genome Analysis (BPGA v. 1.3) [75] for handling the computations. The obtained data showed that contribution of the core genome increased to 84.7% (3726 genes) while the accessory and unique pangenome fractions shrank to either 7.2% (318 genes) or 8.1% (356 genes) of the whole *D. solani* pangenome (4400 genes) as shown in Fig. 2a and Table 3. A reduction in the pool of unique genes was expected due to the larger number of genomic sequences considered. Similarly, the higher quality of genomes used here (as complete genomes) could likely have produced a better assignment of orthologs than in the previous study. However, we cannot a priori exclude a possibility that the use of different software for computing the pangenome between the two studies could have influenced the results.

**Table 3:** Pangenome statistics for 22 *Dickeya solani* genomes

***D. solani*** genome	Pangenome			
	Core Genes	Accessory Genes	Unique genes	Absent Genes
**IFB0167**	**3726**	**256**	**0**	**0**
**IFB0212**	**3726**	**254**	**0**	**0**
**IFB0231**	**3726**	**256**	**0**	**0**
**IFB0311**	**3726**	**250**	**0**	**1**
**IFB0417**	**3726**	**256**	**17**	**5**
**IFB0421**	**3726**	**255**	**4**	**1**
**IFB0487**	**3726**	**237**	**20**	**27**
**IFB0695**	**3726**	**232**	**5**	**19**
IFB0099	3726	255	0	0
IFB0158	3726	261	1	0
IFB0221	3726	261	0	0
IFB0223	3726	249	0	3
IPO 2222	3726	271	2	0
GBBC 2040	3726	219	10	24
MK10	3726	260	5	2
MK16	3726	260	0	0
D s0432–1	3726	262	1	0
PPO 9019	3726	258	2	0
PPO 9134	3726	258	1	0
RNS 05.1.2A	3726	113	286	107
RNS 07.7.3B	3726	254	0	0
RNS 08.23.3.1A	3726	255	2	0

The presented data were calculated with the use of BPGA software [75]. The genomes depicted in bold have been de novo sequenced and assembled in the frames of this research. The included reference genomes have been annotated with the use of Prokka [68] prior to conducting the pangenome analysis

Details on the contribution of specific *D. solani* genomes to the pangenome of this species are depicted in Table 3. The number of accessory genes detected in specific *D. solani* genomes ranged from 113 (RNS 05.1.2A) to 271 (IPO 2222). Regarding unique genes, there were nine strains deprived of such features (IFB0099, IFB0167, IFB0212, IFB0221, IFB0223, IFB0231, IFB0311, MK16, RNS 07.7.3B), in contrast to RNS 05.1.2A possessing even 286 unique genes (Table 3). Thirteen of the *D. solani* strains included, i.e. IFB0099, IFB0158, IFB0167, IFB0212, IFB0221, IFB0231, IPO 2222, MK16, D s0432–1, PPO 9019, PPO 9134, RNS 07.7.3B and RNS 08.23.3.1A, did not contain any genes stated as absent, contrary to RNS 05.1.2A strain, which lacked a huge number of 107 genes present in the other genomes analysed (Table 3). Construction and extrapolation of the core- and pan-genome plots (Fig. 2B), calculated with the use of the exponential curve fit model and power-law regression model, respectively, revealed that with the b parameter equalling 0.0256574, the pangenome of *D. solani* has been almost closed. In other words, the unique gene pool should no longer expand by addition of newly sequenced *D. solani* genomes.

### Functional assignment of the *D. solani* pangenome fractions

**Fig. 3:**
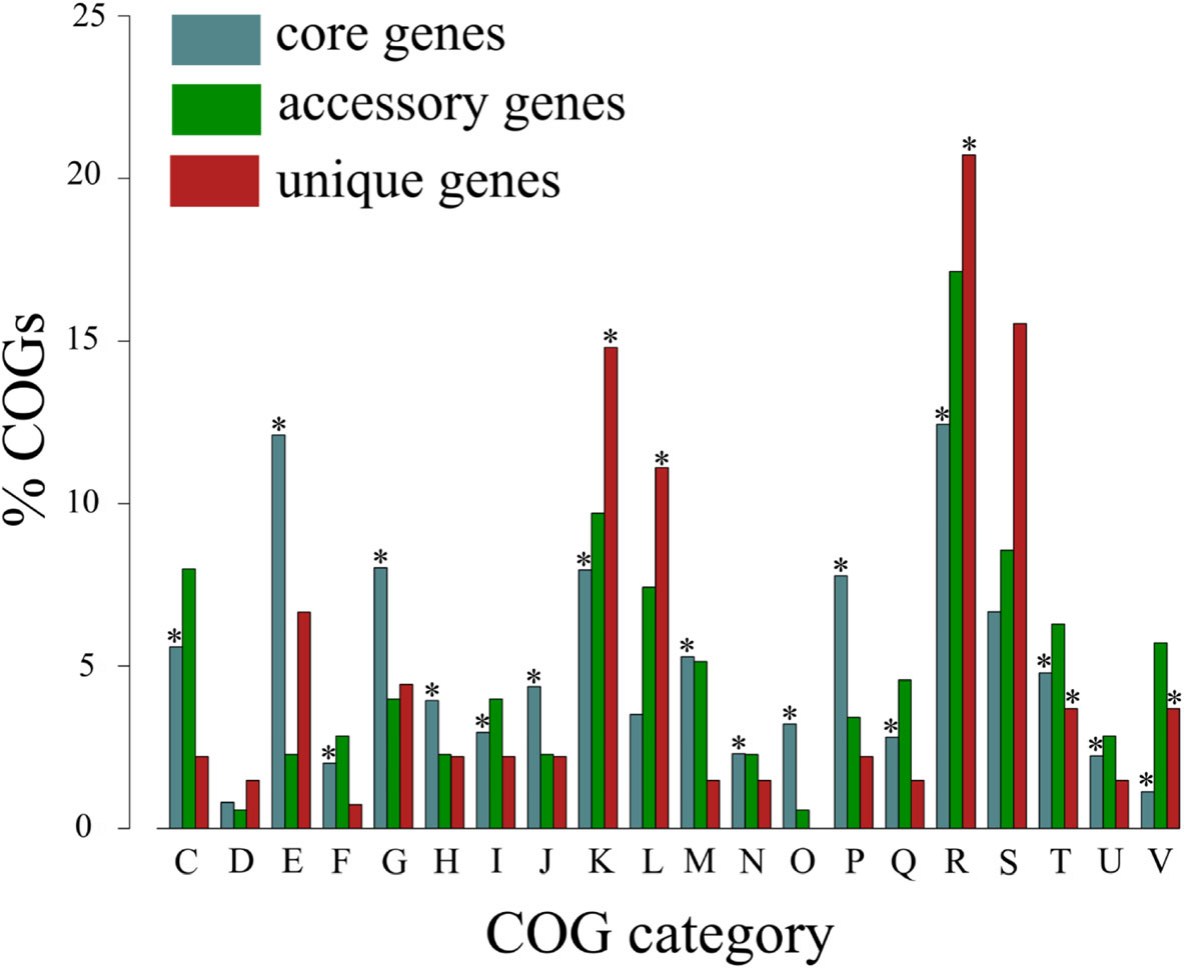
Functional assignment of *Dickeya solani* pangenome fractions. Comparative functional analysis was performed by mapping of the core (blue), accessory (green) and unique (red) genes to the following COG categories: C - energy production and conversion, D - cell cycle control, cell division, chromosome partitioning, E - amino acid transport and metabolism, F - nucleotide transport and metabolism, G - carbohydrate transport and metabolism, H - coenzyme transport and metabolism, I - lipid transport and metabolism, J - translation, ribosomal structure and biogenesis, K - transcription, L - replication, recombination and repair, M - cell wall/membrane/envelope biogenesis, N - cell motility, O - post-translational modification, protein turnover and chaperones, P - inorganic ion transport and metabolism, Q - secondary metabolites biosynthesis, transport and catabolism, R - general function prediction only, S - function unknown, T - signal transduction mechanisms, U - intracellular trafficking, secretion and vesicular transport and V - defence mechanisms, with the use of BPGA v. 1.3 Pan Genome Functional Analysis module [75]. Overrepresented core, accessory and unique COGs within the attributed functional groups are marked with an asterisk (hypergeometric test; *p* < 0.05)

The outcomes of the attribution of the Clusters of Orthologous Groups (COGs) functional categories to the core, accessory and unique gene pools of 22 *D. solani* strains are depicted in Fig. 3. It might be noted that the core pangenome fraction is most abundantly represented in the general function prediction only (R), followed by amino acid transport and metabolism (E), carbohydrate transport and metabolism (G), transcription (K) and inorganic ion transport and metabolism (P) functional groups (Fig. 3). Regarding the accessory pangenome section, after the genes of general function prediction only (R), the ones involved in transcription (K) were highly represented, next these of function unknown (S), engaged in energy production and conversion (C) in addition to replication, recombination and repair (L), however all these overrepresentations were not statistically significant (Fig. 3). In the case of unique genes, they have been assigned most frequently to general function prediction only (R), function unknown (S), transcription (K), replication, recombination and repair (L) and amino acid transport and metabolism (E) COG categories (Fig. 3). Among the above-mentioned functional groups, just overrepresentations of unique COGs within the function unknown (S) and amino acid transport and metabolism (E) categories were not statistically significant. It is worth to keep in mind that a significant number of general function prediction only (R) and function unknown (S) COG categories attributed to the genes from the unique *D. solani* pangenome fraction (Supplementary Table 4) by BPGA v. 1.3 belongs now to the X group i.e. mobilome: prophages, transposons. The groups in which both accessory and unique pangenome fractions dominated in contrast to the core section included general function prediction only (R), function unknown (S), transcription (K), replication, recombination and repair (L) and defence mechanisms (V) classifications (Fig. 3).

It needs to be considered that the number of attributed core COGs was 3300, while the number of accessory and unique COGs equalled 157 and 120, respectively. The largest number of the assigned unique COGs derived from the genome of RNS 05.1.2A (106) followed by IFB0487 (8), IFB0417 (4) IFB0421 (1) and MK10 (1) (Supplementary Table 4). Among the functional roles of the assigned unique *D. solani* COGs, it is worth noticing, for instance, the genes encoding numerous transcriptional regulators (e.g. AcrR, ArsR, LysR, MarR, RpiR, AlpA, DksA), chemotaxis and adhesion proteins, ABC-type transport system components, proteins engaged in the stress response system (alkylhydroperoxidase, SbcCD, LexA), non-ribosomal peptide synthetase, components of the toxin-antitoxin system (RelBD), efflux permeases (MRS), DNA modification methylases, exo and endonucleases, mobile elements (transposase InsO), in addition to abundant prophage-associated proteins (e.g. tail protein, integrase, portal protein BeeE, primase, protein D, protein U, repressor protein C, protein W, protein X, DNA circulation protein, terminase-like protein, capsid-like protein, YmfQ, head maturation protease, head-tail adaptor) (Supplementary Table 4).

### Core-genome-based phylogeny on *D. solani* strains

**Fig. 4:**
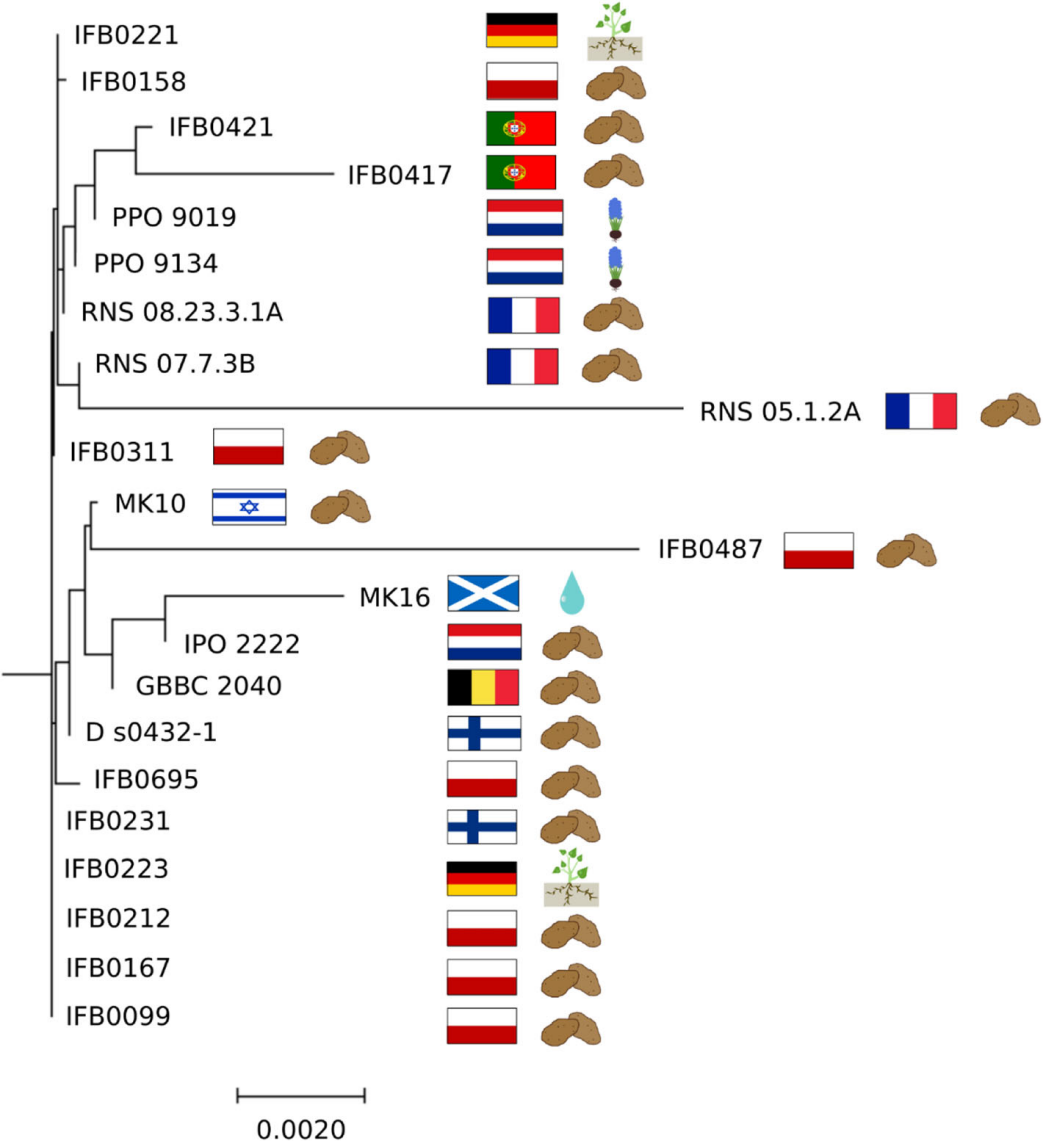
Phylogenetic analysis of the studied *Dickeya solani* strains based on concatenated core gene alignments. ‘Species phylogenetic analysis’ functional module of BPGA v.1.3 [75] software was utilized for generation of a neighbour-joining phylogenetic tree. The tree scaling is based on the distance matrix. Flags mark the countries of origin of the included *D. solani* strains. Also schematic representation of the environmental source (potato tissue, hyacinth tissue, river water or potato rhizosphere) of each *D. solani* strain is shown

To the best of our knowledge, this is the first report on a large-scale genome-wide evolutionary study involving 22 *D. solani* strains. In the first large clade of the generated neighbour-joining phylogenetic tree computed on concatenated core gene alignments (Fig. 4), two strains obtained from Portugal (IFB0417, IFB0421) grouped in proximity to the ones isolated from hyacinths in the Netherlands (PPO 9019, PPO 9134). The above-listed strains are depicted in a subclade also with *D. solani* strains isolated in France (RNS 05.1.2A, RNS 07.7.3B, RNS 08.23.3.1A), in addition to IFB0158 strain isolated in Poland that grouped closely to IFB0221 strain from Germany. All before-mentioned strains are hypothesized to share a common ancestor with IFB0311 from Poland that is the last strain included in the first large clade. To start with the second large clade, there is a MK16 strain from Scotland assembled together with the *D. solani* type strain IPO 2222 from the Netherlands. It is especially intriguing taking into account that Scotland produces 99.5% of its seed potato tubers, while the Netherlands is a potent exporter of this material with strict certification policies [21]. These two *D. solani* strains share a most recent common ancestor with GBBC 2040 from Belgium, which nicely coincides with the fact that Belgium imports huge amounts of seed potatoes, mainly from the Netherlands, followed by France, Germany and Denmark (https://www.trademap.org/; accessed 18.03.2020). The three above-mentioned strains have the same most recent common ancestor as MK10 from Israel grouped together with IFB0487 isolated in Poland in 2013. These two subclades share a common progenitor with D s0432–1 from Finland, and the previous ancestor with IFB0695 from Poland. The two above-described large clades have most recent common ancestors with the pool of closely related strains: IFB0231 (from Finland), IFB0223 (from Germany), IFB0212 (from Poland), IFB0167 (from Poland) and IFB0099 (from Poland) (Fig. 4). It might be spotted that the trade routes of seed, industrial and table potatoes find some reflection in the computed phylogeny.

Taking into consideration that the applied BPGA v. 1.3 software extracts protein sequences (excluding paralogs) from 20 random orthologous gene clusters to generate core genome-based phylogeny (Fig. 4), the herein presented visualization might give a hint on the evolutionary relatedness between the studied *D. solani* strains, but putatively shall not provide conclusive results. Rather, no obvious correlation between the geographical origin of the strains and the computed genome-wide relationships profiting from the core fraction was observed. However, if we have a look at the lengths of the tree branches reflecting the calculated distances, the recognizable divergence of RNS 05.1.2A strain, followed by IFB0487, MK16 and IFB0417 might be spotted (Fig. 4).

## Discussion

Among achievements of this work, one should notice further proofs for high potency of the herein applied genome assembly pipeline described for the first time in our former work [31]. Out of 8 *D. solani* genomes sequenced de novo in this study, 4 have been closed to a full chromosome and the remaining ones contained just 2–7 scaffolds. It is worth underlining that solely PacBio RSII reads have been used during the assembly process, by these means lowering the required financial effort associated with additional acquisition of MiSeq Illumina reads. Furthermore, all the herein utilized software is open-source, contrary to for instance CLC Genomics Workbench v5 utilized by Pedron et al. [47] for assembling the Illumina HiSeq 2000 reads of *D. solani* RNS 08.23.3.1A strain into 42 contigs with N_50_ of 299,659. Interestingly, the sequence of RNS 08.23.3.1A was later on improved by Khayi et al., (2014) [60] into a fully closed chromosome containing the N bases by application of scaffolding, home-made scripts in addition to Sanger sequencing of the PCR amplicons. The herein utilized approach is less laborious and does not require significant bioinformatic skills.

Notably, a remarkable progress has been made on the assembling of *D. solani* genomic sequences in the recent years. For instance, a draft genome of *D. solani* IFB0099 reported before [76] consisted of 97 contigs. This sequence was assembled with Celera from 454 pyrosequencing and PacBio reads after trimming with StreamingTrim software [77]. The resulting assembly contained 5,094,121 bp (%GC 56.40), exceeding by 161,201 bp the improved closed circular genome of IFB0099 (%GC 56.24) obtained with the use of the developed genome assembly pipeline [31]. In spite of the same annotation software utilized, the total number of protein-coding genes, i.e. 4365 [76] vs. 4164 [31], in addition to the number of tRNA- or rRNA-encoding sequences, i.e. 129 [76] vs. 97 [31], varied considerably between the above-mentioned versions, which points to the crucial importance of obtaining high quality genomic assemblies prior to undertaking any comparative genomic analyses.

An alternative approach to assembling of *D. solani* genomes was undertaken by Garlant et al. [38]. The reads for D s0432–1 strain were acquired with Roche 454 GS Flx Titanium chemistry and assembled by using Newbler that generated 98 contigs. Gaps in this assembly were filled in by sequencing PCR or linker-PCR products using an ABI 3730 capillary sequencer. Final gap closing involved the Gap4 program (Staden package). This laborious and costly approach yielded a genome consisting finally of 4 contigs, which discloses obvious benefits of the herein utilized genome assembly pipeline. Another strategy was chosen by Pritchard et al. [44] that assembled 4 *D. solani* genomes into 23–224 scaffolds by relying on 454 pyrosequencing (3 genomes - MK10, MK16, IPO 2222) or IlluminaGAIIx (1 genome - GBBC 2040) technologies. The genome of IPO 2222 was assembled de novo with the use of 454 Life Sciences Newbler v2.5.3. In the case of MK10 and MK16, meta-assembly of Newbler de novo and reference-guided assemblies to the IPO 2222 reference genome were performed. Regarding a GBBC 2040 genome, for which solely Illumina reads have been acquired, CLC bio assembly module was implemented for mapping the reads to the IPO 2222 reference genome [44]. The N_50_ values reported for the first released versions of the above-mentioned genomes were much lower (from 40,901 to 485,700, for GBBC 2040 and MK16, respectively [44]) than in the case of the revised versions included here as references. The assemblies reported by Pritchard et al. [44] have been further improved since their release, reaching in the herein utilized versions 1–3 contigs, though the number of the incorporated N bases (2100–27,548) is still quite large. It is worth noticing that the previous assemblies differ significantly from the following ones regarding for instance the genome length. In the work of Pritchard et al. [44], IPO 2222 was reported to possess 4,857,348 bp, the version of this genome included in the here-presented research exhibits 4,867,258 bp, while the length of the one that might be currently (13.02.20) downloaded from the Genbank database equals 4,919,833 bp. This further proves the importance of obtaining high quality assemblies before conducting any genomic comparisons.

One of the reasons behind undertaking search for plasmids in draft genomic sequences of *D. solani* is that the occurrence of such extrachromosomal molecules might be an explanation for the contig-based status of the assembly. Our data confirmed that up to the present day solely one plasmid sequence has been described in the *D. solani* species, namely the one harboured by PPO 9019 strain isolated from hyacinth [61]. Notably, this extrachromosomal genetic sequence shared complete identity (100%) with the plasmid of *Burkholderia ambifaria* AMMD (CP000443.1) [39]. In spite of sharing a common plasmid, there is another argument pointing to the association between *D. solani* and *B. ambifaria*, as these two species exhibited notable similarities in the O-polysaccharides (OPS) within their lipopolysaccharide (LPS) structures [78, 79]. In more detail, 6-deoxy-D-altrose that was found in the OPS of *D. solani* and *D. dadantii* [78] was up to now reported only as a constituent of a disaccharide repeating unit →4)-α-d-Rhap-(1 → 3)-β-d-6dAltp-(1 → in the OPS of *B. ambifaria* type strain LMG 19182 [79]. Interestingly, *B. ambifaria* was noted to possess two diverse OPS molecules, which might be related with the adaptation of these strains to various environmental niches such as plant leaves, roots and rhizospheres, forest soil or even sputum or respiratory tract of patients suffering from cystic fibrosis [80]. Specifically, the *B. ambifaria* LMG 19182 strain was isolated from the rhizosphere of pea in Wisconsin (USA) in 1985 [81]. As suggested previously, sugar composition of O-antigen follows the availability of monosaccharide substrates [82], therefore the occurrence of D-altrose in the OPS of plant-associated isolates of *Dickeya* spp. and *B. ambifaria,* together with the previous proofs for horizontal gene transfer (HGT) resulting from plasmid transmission between these species [39], gives a clue about their coexistence in natural environment.

In this work, 22 whole genomic sequences of *D. solani* strains were juxtaposed, which revealed extraordinarily high level of homogeneity within this species. Several previous studies also focused on whole genome comparisons that included *D. solani* chromosomal sequences, but a significantly lesser number of genomes and other bioinformatics software were utilized. Pedron et al. [47] juxtaposed the genome of *D. solani* 3337 to the one of *D. dadantii* 3937 with the use of Mauve. In spite of a high level of synteny between these genomes, there were noted two insertions and a notable inversion between two *rrs* ribosomal RNA-encoding operons [47]. Interestingly, des Essarts et al. [59] spotted two syntenic disruptions and a notable evidence for horizontal gene transfer in the genome of *D. solani* 3337 in contrast to *D. dianthicola* RNS04.9. The scale of study has been enlarged in the work of Garlant et al. [38], in which the genomic sequence of *D. solani* D s0432–1 was compared with a pool of representative genomes of other *Dickeya* spp. i.e. *D. dadantii* 3937, *D. zeae* Ech586, *D. paradisiaca* Ech703 and *D. chrysanthemi* Ech1591. The lowest number of rearrangements was observed between *D. solani* D s0432–1 and *D. dadantii* 3937 [38]. Subsequently, Khayi et al. [39] reported that the genomes of two *D. solani* strains, namely 3337 and 0512, exhibit significant syntenic conservation accordingly to Mauve-based visualization. In our previous work [31], the same bioinformatic tool was incorporated to prove the lack of significant chromosomal rearrangements in the closed genomes of 5 *D. solani* strains. In more detail, the presence of 3 syntenic blocks was revealed in that work with two inversions regarding IFB0099, IFB0223 and RNS 08.23.3.1A strains, contrary to GBBC 2040 and IPO 2222 [31].

Additional evidence for a notable uniformity among the analysed *D. solani* genomes was provided by the herein computed pairwise comparisons relying on ANIb (98.55–100), ANIm (98.71–99.99) and Tetra correlation (0.99976–1.0) values. All outcomes of the ANI-based calculations easily exceeded the 95–96% species delineation threshold that corresponds to 70% DDH [83]. Previously, Garlant et al. [38] juxtaposed the genome of *D. solani* D s0432–1 to several other members of the *Dickeya* genus, i.e. *D. dadantii* 3937, *D. zeae* Ech586, *D. paradisiaca* Ech703 and *D. chrysanthemi* Ech1591, with the resultant ANI values of 94, 85, 79 and 86%, respectively [38]. The work of des Essarts et al. [59] further supported the closest relationship between *D. solani* (3337 strain) and *D. dadantii* (3937 strain) with ANI and DDH values of 94 and 55%. Even though the herein investigated *D. solani* genomes turned out to be highly homogenous basing on ANI calculations as it was suggested previously [39, 60], the computed values did not always exceed the 99.9 threshold demonstrated before [39, 58, 84]. However, the gathered data are in agreement with our former study [31], in which the ANI values determined for pairwise comparisons among 14 *D. solani* genomes ranged from 98.60 to 99.99%. It is worth keeping in mind that often various software has been utilized for ANI calculations e.g. nucmer with script calculate_ani.py [84, 85], ChunLab’s online Average Nucleotide Identity Calculator (EzBioCloud) [31, 86] or JSpecies [38, 47], which might be the cause of slight discrepancies in the reported genome-to-genome deviations between *D. solani* strains.

The fact that the genome of *D. solani* RNS 05.1.2A clearly stood out from the other analysed both in terms of the genomic structure and the computed pairwise-comparisons is putatively associated with the abundance of unique genes as further proven in the pangenome-related section and suggested in the former studies [31, 39]. Besides, modest dissimilarities in comparison to the included genomic pool were noted for RNS 07.7.3B, PPO 9019 and PPO 9134 sequences, which were also reported to show discrepancies in SNPs/InDels in contrast to other *D. solani* genomes [58]. Khayi et al. [39] postulated HGT from a closely related habitant of the same ecological niche, namely *D. dianthicola*, as a possible explanation for this phenomenon. The fact that both PPO 9019 and PPO 9134 strains were acquired from hyacinths and stood out solely in the ANI calculations, in contrast to the computed correlation indexes of the tetra-nucleotide signatures, might be a point in favour of the HGT-based hypothesis.

Moving to the outcomes of pangenome-oriented analyses, the whole gene pool of 22 *D. solani* strains comprises 4400 genes divided between large core (84.7%) in addition to significantly smaller accessory (7.2%) and unique (8.1%) pangenome fractions. Importantly, with the applied number of whole genomic sequences, the almost closed status of the *D. solani* pangenome has been reached. Such a feature is regarded as characteristic of the taxa whose representatives either occupy isolated ecological niches or lack efficient mechanisms for gene exchange and recombination [87]. Therefore, *D. solani* joined the group of real specialized pathogens with closed pangenomes [88] including e.g. *Bacillus anthracis* [62], *Mycobacterium tuberculosis* [89], *Clostridium difficile* [90], *Yersinia pestis* [91] or *Staphylococcus aureus* [92]. In contrast to *D. solani*, we reported that another member of the *Pectobacteriaceae* family, namely *Pectobacterium parmentieri*, possesses an open pangenome [93]. Computation with the use of Roary on 15 *P. parmentieri* genomes disclosed a notably lesser contribution of core (52.8%) and higher of accessory (20.9%) and unique (26.3%) pangenome fractions in comparison to *D. solani*. We associated the overrepresentation of the dispensable pangenome part with high genomic plasticity of *P. parmentieri* [93], suggesting a less clonal population structure with respect to that of *D. solani* [19, 21, 23, 59]. Thus, the closely related *P. parmentieri* species adhered to the categories of non-specialized species or opportunistic pathogens that often exhibit open pangenomes [88, 94] along with, for instance, *Escherichia coli* [95], *Streptococcus agalactiae* [96], *Listeria monocytogenes* [97], *Legionella pneumophila* [98] or *Salmonella typhi* [99]*.* One should bear in mind that the closed/open pangenome status of a species might have been affected by the number and representativeness of the genomes selected for the analysis [94]. Besides, not without importance is the software utilized for performing the pangenome calculations.

Attribution of the genes originating from diverse *D. solani* pangenome fractions to functional COG categories showed overrepresentation of phage/mobile elements- and transcription- associated groups in the studied accessory and unique pangenome parts. Notably, the genome of RNS 05.1.2A (followed by the sequences of IFB0487 and IFB0417 strains) had the most significant contribution to this phenomenon. Similarly to this research, our previous study [31] pointed to RNS 05.1.2A genome as the most distant from the others tested, basing on the largest number of unique genes as calculated by Mauve. The MK10 genome was also mentioned in the former study as a divergent one [31], although in the current work it possessed solely 1 unique COG, just as IFB0421.

The herein described high abundance of phage/mobile elements-related COG classifications in the accessory and unique *D. solani* gene pools is in agreement with our previous study [31] which underlined the importance of prophages in the evolution of *D. solani* genomes. Out of 35 prophage sequences detected in 14 *D. solani* genomes, the majority of the strains harbored 2–3 prophages with the exception of RNS 05.1.2A, which showed the presence of 7 such prophage-like elements [31]. Also Khayi et al. [39] reported the RNS 05.1.2A strain to possess unique phage elements and hypothetical or unknown proteins except for some other strain-specific genes coding for two putative ABC transporters, two hypothetical virulence factors and one methyl-accepting chemotaxis protein, similarly to the types of COGs that have been established in the unique pangenome fraction described in the current research. It is also worth noticing that a protein family involved in adhesion has been spotted in the *D. solani* unique pangenome fraction, which is in accordance with our previous suggestions [31] on the putative involvement of these proteins in the overall *D. solani* virulence. Furthermore, quite a big number of the observed transcription-associated unique COGs confirms the assumptions of Potrykus et al. [32, 33] on the correlation between the regulation of genes expression and the noted differences in the virulence of various *D. solani* strains.

In the current study, the first computed core genome-based phylogeny on 22 *D. solani* strains showed no obvious correlations between the geographical origin of the isolates and their evolutionary relationships. Though, the calculated evolutionary distances pointed to notable divergence of RNS 05.1.2A strain, similarly to what was observed in the other herein conducted comparative genomic analyses. In accordance to our data, an outgrouping of RNS 05.1.2A strain was described previously by Khayi et al. [58] basing on a phylogenetic tree computed on *gapA* sequences. Also MLST analysis on the concatenated sequences of *rpoD*, *gyrB*, *recA*, *rpoS*, *dnaX*, *dnaA*, *gapA*, *fusA*, *rplB*, *purA* and *gyrA* housekeeping genes differentiated RNS 05.1.2A from the other *D. solani* isolates included [39]. One should bear in mind that the search for phylogenetic relations among *D. solani* strains is impaired to some degree by high similarity of these isolates and their tendency to group together regardless of the origin and year of isolation. Such a phenomenon was reported before by van der Wolf et al. [19] basing on the phylogenies computed on PFGE profiles, IGS regions, single house-keeping genes, i.e. *dnaX, recA, dnaN, fusA, gapA, purA, rpoS, rplB* or the concatenated sequences of all the above-listed 8 genes in addition to IGS. In that study, solely the fatty acids fingerprints showed subtle differences between *D. solani* strains. It seems that phylogenetic relatedness between diverse strains is affected to some extent by the applied marker and bioinformatic method, indicating that the most appropriate approach to be used still has to be revealed.

## Conclusions

In view of a high need for extensive comparative genomics studies conducted on the economically important members of the *Pectobacteriaceae* family [100], at first we decided to enlarge the available pool of *D. solani* genomes, taking into consideration that this species was pointed to as a significant threat to potato production in Europe [21]. 8 novel *D. solani* genomes have been sequenced and assembled either to the closed genomes or high-quality draft-status assemblies containing just a few contigs. An exceptionally high level of homogeneity among 22 *D. solani* genomes was proven in whole-genome comparison, ANIb, ANIm, Tetra and pangenome-oriented analyses. Notably, the genome of *D. solani* RNS 05.1.2A stood out from the others tested in all the above-mentioned calculations. After the inclusion of 22 *D. solani* genomes, the pangenome of this species consisting in 84.7% of core, 7.2% of accessory and 8.1% of unique genes, turned out to be almost closed. The assignment of the genes included in the *D. solani* pangenome fractions to functional COG categories revealed that higher percentages of accessory and unique pangenome parts in contrast to the core section are encountered in phage/mobile elements- and transcription- associated groups, with the genome of RNS 05.1.2A strain having the most significant contribution to this phenomenon. The first large-scale genome-wide phylogenetic study based on concatenated core gene alignments showed rather no obvious correlations between the geographical origin of the strains and the computed evolutionary relationships which might reflect to some point the specificity of the international seed potato market.

## Methods

### Collection and identification of *D. solani* strains

Out of 8 *D. solani* strains subjected to de novo whole-genome sequencing within the frames of this study (Table 1), 7 (IFB0167, IFB0212, IFB0311, IFB0417, IFB0421, IFB0487, IFB0695) have been isolated and identified to the species level by our research group. The implemented methods have been described previously [27, 101]. Briefly, symptomatic potato tissue has been collected from seed potato fields (either in Poland or Portugal; Table 1), homogenized in phosphate buffer, serially-diluted in 0.85% NaCl and plated on semiselective Cristal Violet Pectate (CVP) medium [102]. Post 48 h incubation at 28 °C, the cavity-forming units were collected and purified to reach the axenic culture state by several replating steps on CVP and TSA media. Isolates belonging to the *Dickeya* genus were identified with the use of PCR either with ADE1 and ADE2 [103] or Df and Dr primers [101, 104]. The isolates have been assigned to *D. solani* species basing on PCR reactions with SOL-C or SOL-D starters [36] and comparison of the sequences of *dnaX* housekeeping gene [23]. All strains were subsequently frozen in 40% glycerol and stored in the collection of phytopathogenic bacteria of Intercollegiate Faculty of Biotechnology University of Gdansk and Medical University of Gdansk for subsequent analyses. IFB0231 strain was isolated in Finland (Table 1) and identified to *D. solani* species as described by Degefu et al. (2013) [28].

### De novo sequencing of *D. solani* genomes

*D. solani* strains designated for de novo sequencing were selected in such a way as to reflect the highest possible diversity among the already studied isolates [27–29] at our possession (Table 1).

Regarding the firstly analysed four strains (IFB0167, IFB0212, IFB0231 and IFB0311), they have been sent in the form of cell pellets to GATC Biotech (Constance, Germany) for DNA isolation, quality control, libraries preparation and sequencing with the use of two platforms, namely PacBio RSII and Illumina MiSeq. After proposal of the PacBio-based optimal genome assembly pipeline for *D. solani* [31], DNA of the latter 4 *D. solani* strains (IFB0417, IFB421, IFB487 and IFB0695) was sequenced at GATC Biotech just on the PacBio RSII platform (Motyka-Pomagruk et al., submitted). Accordingly to the genome assembly pipeline described in our previous work [31], *D. solani* genomic sequences have been assembled from solely PacBio RSII reads. At first, these raw reads were filtered from adapters with the use of SMRT Analysis software (Pacific Biosciences, USA). The coverage of the filtered reads in terms of IFB0167, IFB0212, IFB0231, IFB0311, IFB0417, IFB421, IFB487 and IFB0695 equalled 274x, 63x, 157x, 57x, 212x, 243x, 211x and 230x, respectively. Then, these reads were corrected, trimmed and assembled with the use of Canu [66]. Getting consensus and variant calling was achieved thanks to Quiver [67], while functional annotation was conducted with Prokka [68] as previously reported [31]. The assembled and annotated de novo sequenced genomes of 8 *D. solani* strains have been deposited in the National Center for Biotechnology Information (NCBI) Genome database (Bioproject no. PRJNA611911) under the accession numbers listed in Table 2.

### Comparative genomics

Beside 8 de novo sequenced genomes (Tables 1 and 2), 14 *D. solani* reference sequences (Table 2) were included in the conducted comparative genomic analyses: IFB0099 (CP024711 [31]), IFB0158 (PENA00000000 [31]), IFB0221 (PEMZ00000000 [31]), IFB0223 (CP024710 [31]), IPO 2222 (AONU01000000 [44]), GBBC 2040 (AONX01000000 [44]), MK10 (AOOP01000000 [44]), MK16 (AOOQ01000000 [44]), D s0432–1 (AMWE01000000 [38]), PPO 9019 (JWLS01000000 [39]), PPO 9134 (JWLT01000000 [39]), RNS 05.1.2A (JWMJ01000000 [39]), RNS 07.7.3B (JWLR01000000 [39]) and RNS 08.23.3.1A (AMYI01000000 [60]). The above-listed reference sequences have been downloaded from the NCBI Genome database in a FASTA format. To assure the uniformity of the attributed genomic annotations, also the reference *D. solani* sequences have been processed with Prokka v. 1.12 [68] software as it was the case of de novo assembled sequences.

The number of contigs in the genomes, %GC in addition to N_50_ and L_50_ metrics were computed with Quast v. 5.0 [105]. The search for plasmid sequences among the draft *D. solani* genomic assemblies was accomplished with PlasmidFinder v. 2.0 [69] with the default settings. Whole genome comparison of 22 *D. solani* sequences has been computed with the use of BRIG v. 0.95 [72]. The included pairwise genome comparisons are based on ANIb, ANIm and computation of the correlation indexes of the tetra-nucleotide signatures by applying JSpecies webserver (accessed 02.2019) [106].

### Pangenome analysis

BPGA v. 1.3 [75] was utilized for pangenome studies in addition to the pangenome-based downstream analyses including core genome phylogeny and functional assignments to the COGs categories. Sequence data were pre-processed and clustered with the use of USEARCH (50% cut off) [107]. Further computation of the output led to the generation of a tab delimited gene presence/absence binary matrix (pan-matrix), utilized for pangenome pattern calculations with iterations (20 as a default). For core genome-based phylogeny, BPGA v. 1.3 [75] extracted protein sequences (excluding paralogs) from 20 random orthologous gene clusters. Then MUSCLE [108] was applied for alignment of concatenated core genes resulting in the construction of a neighbour-joining phylogenetic tree. Last but not least, USEARCH [107] was implemented for functional assignments on the basis of the best hits with the reference COG database [109]. COG IDs were attributed to all representative protein sequences from each orthologous protein cluster based on the BLAST algorithm [110]. Percentage occurrences of the assigned COG categories were calculated by BPGA v. 1.3. Overrepresentation of core, accessory and unique COGs within the attributed functional groups was assessed by a hypergeometric test at *p* < 0.05 in R v. 3.1.3. In addition, the COG IDs assigned to *D. solani* unique COGs were manually searched against the COG database [109] for stating the up-to-date functions played by the individual protein family clusters.

## Supplementary information

**Additional file 1: Table S1.** ANIb values calculated for the studied *Dickeya solani* genomes. Description of data: BLAST calculation of ANI (ANIb) was performed with the use of JSpecies [106]. The upper number refers to the ANIb value while the lower depicted in parentheses is the percentage of the aligned sequences.

**Additional file 2: Table S2.** ANIm values calculated for the studied *Dickeya solani* genomes. Description of data: MUMmer calculation of ANI (ANIm) was performed with the use of JSpecies [106]. The upper number refers to ANIm value while the lower depicted in parentheses is the percentage of the aligned sequences.

**Additional file 3: Table S3.** Correlation indexes of the tetra-nucleotide signatures computed for the studied *Dickeya solani* genomes. Description of data: Computation of the correlation indexes of the tetra-nucleotide signatures was conducted with JSpecies [106].

**Additional file 4: Table S4.** The functions of the unique *Dickeya solani* COGs. Description of data: ^a^ The first category was attributed by the BPGA v. 1.3 software. If it differed from the category currently allocated to certain COG IDs in the COG database, the up-to-date assignment after the slash mark is depicted. COG categories: A - RNA processing and modification, B - chromatin structure and dynamics, C - energy production and conversion, D - cell cycle control, cell division, chromosome partitioning, E - amino acid transport and metabolism, F - nucleotide transport and metabolism, G - carbohydrate transport and metabolism, H - coenzyme transport and metabolism, I - lipid transport and metabolism, J - translation, ribosomal structure and biogenesis, K - transcription, L - replication, recombination and repair, M - cell wall/membrane/envelope biogenesis, N - cell motility, O - post-translational modification, protein turnover and chaperones, P - inorganic ion transport and metabolism, Q - secondary metabolites biosynthesis, transport and catabolism, R - general function prediction only, S - function unknown, T - signal transduction mechanisms, U - intracellular trafficking, secretion and vesicular transport, V - defence mechanisms, W - extracellular structures, X - mobilome: prophages, transposons, Y - nuclear structure and Z – cytoskeleton.

## Data Availability

**De novo*****sequenced D. solani genomes.*** Data generated in this whole genome sequencing project are available in the Genome database of the National Center for Biotechnology Information (NCBI) under the BioProject PRJNA611911. The analysed *D. solani* strains have been attributed with the following Biosample nos. SAMN14352303, SAMN14352304, SAMN14352305, SAMN14352306, SAMN14352307, SAMN14352308, SAMN14352309 and SAMN14352310. The assembled and annotated full genomic sequences of the herein de novo sequenced *D. solani* genomes are available in the NCBI Genome database under the following accession nos: CP051457, JABAON000000000, CP051458, JABAOO000000000, CP051459, CP051460, JABAOP000000000 and JABAOQ000000000 (Table 2). The datasets generated and/or analyzed during this study will not be publicly available prior to first publication of the herein presented manuscript. After publication, the datasets will be available from the corresponding author on a reasonable request. ***The reference D. solani genomes.*** The included 14 reference *D. solani* genomic sequences have been downloaded in a FASTA format from the NCBI Genome database, in which they have been deposited under the following accession nos. CP024711, PENA00000000, PEMZ00000000, CP024710, AONU01000000, AONX01000000, AOOP01000000, AOOQ01000000, AMWE01000000, JWLS01000000, JWLT01000000, JWMJ01000000, JWLR01000000 and AMYI01000000 (Table 2).

## Abbreviations

ANI: Average Nucleotide Identity
ANIb: BLAST+ calculation of ANI
ANIm: MUMmer calculation of ANI
BRIG: BLAST Ring Image Generator
BPGA: Bacterial Pan Genome Analysis
CVP: Cristal Violet Pectate
COGs: Clusters of Orthologous Groups
DDH: DNA-DNA hybridization
ERIC: Enterobacterial Repetitive Intergenic Consensus
HGT: Horizontal gene transfer
IGS: Intergenic spacer
LPS: Lipopolysaccharide
MALDI-TOF MS: Matrix-Assisted Laser Desorption Ionization Time-Of-Flight Mass Spectrometry
NCBI: National Center for Biotechnology Information
OPS: O-polysaccharides
PFGE: Pulse Field Gel Electrophoresis
PCWDEs: Plant cell wall degrading enzymes
REP-PCR: Repetitive Extragenic Palindromic-PCR
Tetra: Tetra-nucleotide signatures
WGS: Whole genome sequences
