# Supplementary material for: Comparative genomics and pangenome-oriented studies reveal high homogeneity of the agronomically relevant enterobacterial plant pathogen Dickeya solani
Source: BMC Genomics. 2020 Jun 29;21:449. doi: 10.1186/s12864-020-06863-w (PMC7325237; doi:10.1186/s12864-020-06863-w)
Supplement: Supplementary file 1 — Additional file 1: Table S1. ANIb values calculated for the studied Dickeya solani genomes. Description of data: BLAST calculation of ANI (ANIb) was performed with the use of JSpecies [106]. The upper number refers to the ANIb value while the lower depicted in parentheses is the percentage of the aligned sequences. [file 12864_2020_6863_MOESM1_ESM.docx]

|  | **IFB0099** | **IFB0158** | **IFB0167** | **IFB0212** | **IFB0221** | **IFB0223** | **IFB0231** | **IFB0311** | **IFB0417** | **IFB0421** | **IFB0487** | **IFB0695** | **IPO 2222** | **GBBC 2040** | **MK 10** | **MK 16** | **D s0432-1** | **PPO 9019** | **PPO 9134** | **RNS 05.1.2A** | **RNS 07.7.3B** | **RNS 08.23.3.1A** |
| --- | --- | --- | --- | --- | --- | --- | --- | --- | --- | --- | --- | --- | --- | --- | --- | --- | --- | --- | --- | --- | --- | --- |
| **IFB0099** | * | 99.99 (99.25) | 100.00 (99.73) | 100.00 (99.72) | 99.98 (99.27) | 100.00 (99.67) | 100.00 (99.71) | 100.00 (99.70) | 99.98 (99.67) | 100.00 (99.73) | 99.95 (98.96) | 99.97 (99.10) | 99.97 (98.31) | 99.97 (96.67) | 99.97 (98.92) | 99.96 (99.06) | 99.95 (99.52) | 99.91 (98.83) | 99.92 (98.95) | 98.63 (94.94) | 99.96 (98.93) | 99.98 (99.65) |
| **IFB0158** | 99.99 (99.76) | * | 99.99 (99.76) | 99.99 (99.77) | 99.99 (99.73) | 99.99 (99.66) | 99.99 (99.76) | 99.99 (99.77) | 99.98 (99.69) | 99.99 (99.76) | 99.95 (99.01) | 99.97 (99.16) | 99.95 (98.84) | 99.95 (97.11) | 99.96 (99.43) | 99.97 (99.51) | 99.97 (99.59) | 99.91 (99.37) | 99.92 (99.41) | 98.64 (95.19) | 99.96 (99.47) | 99.98 (99.69) |
| **IFB0167** | 100.00 (99.78) | 99.99 (99.26) | * | 100.00 (99.74) | 99.99 (99.30) | 100.00 (99.72) | 100.00 (99.76) | 100.00 (99.74) | 99.98 (99.72) | 100.00 (99.78) | 99.95 (99.01) | 99.97 (99.14) | 99.97 (98.25) | 99.97 (96.51) | 99.97 (98.85) | 99.96 (99.02) | 99.95 (99.57) | 99.91 (98.82) | 99.92 (98.98) | 98.65 (94.86) | 99.96 (98.94) | 99.98 (99.73) |
| **IFB0212** | 100.00 (99.76) | 99.99 (99.29) | 100.00 (99.76) | * | 99.98 (99.29) | 100.00 (99.69) | 100.00 (99.75) | 100.00 (99.72) | 99.98 (99.68) | 100.00 (99.75) | 99.96 (99.01) | 99.98 (99.10) | 99.97 (98.39) | 99.96 (96.63) | 99.97 (98.95) | 99.97 (99.21) | 99.97 (99.54) | 99.91 (98.97) | 99.92 (99.10) | 98.66 (94.94) | 99.96 (99.09) | 99.98 (99.69) |
| **IFB0221** | 99.99 (99.71) | 99.99 (99.68) | 99.99 (99.71) | 99.99 (99.72) | * | 99.99 (99.62) | 99.99 (99.71) | 99.99 (99.71) | 99.97 (99.64) | 99.99 (99.71) | 99.95 (98.94) | 99.97 (99.11) | 99.96 (98.80) | 99.95 (97.16) | 99.96 (99.34) | 99.96 (99.45) | 99.96 (99.53) | 99.90 (99.34) | 99.91 (99.39) | 98.61 (95.14) | 99.95 (99.42) | 99.98 (99.63) |
| **IFB0223** | 100.00 (99.79) | 99.99 (99.29) | 100.00 (99.79) | 100.00 (99.75) | 99.98 (99.32) | * | 100.00 (99.79) | 100.00 (99.75) | 99.98 (99.71) | 100.00 (99.79) | 99.94 (99.04) | 99.98 (99.16) | 99.97 (98.22) | 99.97 (96.53) | 99.96 (98.89) | 99.97 (99.04) | 99.93 (99.60) | 99.91 (98.83) | 99.92 (98.96) | 98.64 (94.85) | 99.96 (98.96) | 99.98 (99.72) |
| **IFB0231** | 100.00 (99.74) | 99.99 (99.25) | 100.00 (99.74) | 100.00 (99.73) | 99.98 (99.27) | 100.00 (99.69) | * | 100.00 (99.70) | 99.98 (99.67) | 100.00 (99.73) | 99.95 (98.96) | 99.97 (99.11) | 99.97 (98.37) | 99.97 (96.72) | 99.97 (98.94) | 99.96 (99.07) | 99.95 (99.53) | 99.91 (98.86) | 99.92 (98.97) | 98.63 (94.95) | 99.96 (98.98) | 99.98 (99.68) |
| **IFB0311** | 100.00 (99.72) | 99.99 (99.31) | 100.00 (99.72) | 100.00 (99.72) | 99.99 (99.31) | 100.00 (99.66) | 100.00 (99.72) | * | 99.98 (99.65) | 100.00 (99.72) | 99.95 (98.95) | 99.97 (99.11) | 99.98 (98.38) | 99.97 (96.66) | 99.97 (98.88) | 99.97 (99.05) | 99.95 (99.53) | 99.91 (98.91) | 99.92 (99.04) | 98.65 (94.93) | 99.96 (99.04) | 99.98 (99.64) |
| **IFB0417** | 99.98 (99.74) | 99.97 (99.25) | 99.98 (99.74) | 99.98 (99.71) | 99.96 (99.25) | 99.98 (99.67) | 99.98 (99.74) | 99.98 (99.70) | * | 99.98 (99.74) | 99.95 (98.99) | 99.97 (99.08) | 99.96 (98.24) | 99.95 (96.54) | 99.95 (98.83) | 99.95 (99.07) | 99.94 (99.54) | 99.89 (98.88) | 99.90 (99.00) | 98.60 (94.90) | 99.94 (98.97) | 99.96 (99.69) |
| **IFB0421** | 100.00 (99.78) | 99.98 (99.21) | 100.00 (99.78) | 99.99 (99.73) | 99.98 (99.24) | 100.00 (99.71) | 100.00 (99.78) | 99.99 (99.74) | 99.98 (99.71) | * | 99.94 (99.03) | 99.99 (99.16) | 99.97 (98.22) | 99.97 (96.47) | 99.96 (98.87) | 99.96 (99.06) | 99.96 (99.58) | 99.91 (98.83) | 99.92 (98.96) | 98.63 (94.91) | 99.96 (98.96) | 99.97 (99.70) |
| **IFB0487** | 99.98 (99.75) | 99.97 (99.19) | 99.98 (99.75) | 99.98 (99.72) | 99.96 (99.18) | 99.98 (99.68) | 99.98 (99.75) | 99.98 (99.72) | 99.97 (99.68) | 99.98 (99.75) | * | 99.95 (99.11) | 99.96 (98.30) | 99.96 (96.55) | 99.95 (98.81) | 99.95 (99.01) | 99.94 (99.54) | 99.89 (98.80) | 99.90 (98.93) | 98.61 (94.84) | 99.94 (98.93) | 99.96 (99.70) |
| **IFB0695** | 99.99 (99.74) | 99.97 (99.27) | 99.99 (99.74) | 99.99 (99.74) | 99.97 (99.27) | 99.99 (99.66) | 99.99 (99.73) | 99.99 (99.73) | 99.98 (99.67) | 99.99 (99.73) | 99.95 (98.98) | * | 99.95 (98.22) | 99.94 (96.54) | 99.96 (98.85) | 99.96 (99.03) | 99.94 (99.54) | 99.90 (98.86) | 99.91 (98.97) | 98.66 (94.78) | 99.95 (99.05) | 99.97 (99.67) |
| **IPO 2222** | 99.99 (98.88) | 99.98 (98.78) | 99.99 (98.90) | 99.99 (98.88) | 99.98 (98.79) | 99.99 (98.82) | 99.99 (98.90) | 99.99 (98.87) | 99.97 (98.83) | 99.99 (98.89) | 99.96 (98.13) | 99.99 (98.25) | * | 99.99 (97.12) | 99.98 (98.77) | 99.99 (98.87) | 99.98 (98.74) | 99.91 (98.73) | 99.92 (98.77) | 98.65 (94.70) | 99.96 (98.81) | 99.98 (98.85) |
| **GBBC 2040** | 99.99 (97.24) | 99.98 (97.13) | 99.99 (97.24) | 99.99 (97.24) | 99.98 (97.13) | 99.99 (97.18) | 99.99 (97.24) | 99.99 (97.24) | 99.97 (97.17) | 99.99 (97.24) | 99.94 (96.53) | 99.98 (96.58) | 99.99 (97.25) | * | 99.97 (97.12) | 99.98 (97.24) | 99.97 (97.11) | 99.90 (97.11) | 99.92 (97.10) | 98.65 (93.51) | 99.96 (97.15) | 99.98 (97.22) |
| **MK 10** | 99.99 (99.46) | 99.98 (99.27) | 99.99 (99.46) | 99.99 (99.46) | 99.98 (99.21) | 99.99 (99.39) | 99.99 (99.46) | 99.99 (99.46) | 99.97 (99.39) | 99.99 (99.46) | 99.96 (98.72) | 99.96 (98.85) | 99.98 (98.85) | 99.97 (97.25) | * | 99.98 (99.42) | 99.97 (99.37) | 99.91 (99.23) | 99.91 (99.25) | 98.62 (94.96) | 99.95 (99.30) | 99.98 (99.44) |
| **MK 16** | 100.00 (99.56) | 99.99 (99.37) | 100.00 (99.56) | 100.00 (99.57) | 99.99 (99.34) | 100.00 (99.47) | 100.00 (99.56) | 100.00 (99.56) | 99.98 (99.50) | 100.00 (99.56) | 99.96 (98.80) | 99.98 (98.91) | 99.98 (98.90) | 99.98 (97.12) | 99.99 (99.34) | * | 99.99 (99.42) | 99.92 (99.34) | 99.92 (99.41) | 98.65 (95.08) | 99.97 (99.43) | 99.99 (99.54) |
| **D s0432-1** | 100.00 (99.77) | 99.99 (99.36) | 100.00 (99.77) | 100.00 (99.77) | 99.99 (99.31) | 100.00 (99.70) | 100.00 (99.77) | 99.99 (99.70) | 99.98 (99.71) | 99.99 (99.77) | 99.95 (99.01) | 99.98 (99.16) | 99.98 (98.40) | 99.98 (96.75) | 99.99 (98.96) | 99.99 (99.15) | * | 99.91 (98.96) | 99.92 (99.05) | 98.67 (94.98) | 99.96 (99.09) | 99.99 (99.74) |
| **PPO 9019** | 99.92 (99.71) | 99.91 (99.56) | 99.92 (99.71) | 99.92 (99.71) | 99.91 (99.53) | 99.92 (99.62) | 99.92 (99.71) | 99.92 (99.69) | 99.90 (99.64) | 99.91 (99.70) | 99.89 (98.92) | 99.89 (99.11) | 99.89 (98.94) | 99.88 (97.29) | 99.88 (99.50) | 99.89 (99.61) | 99.87 (99.55) | * | 99.84 (99.67) | 98.58 (95.37) | 99.88 (99.71) | 99.90 (99.67) |
| **PPO 9134** | 99.93 (99.61) | 99.92 (99.42) | 99.93 (99.61) | 99.93 (99.62) | 99.92 (99.40) | 99.93 (99.53) | 99.93 (99.61) | 99.93 (99.61) | 99.91 (99.54) | 99.93 (99.61) | 99.89 (98.84) | 99.90 (99.00) | 99.91 (98.74) | 99.90 (96.99) | 99.89 (99.35) | 99.90 (99.49) | 99.89 (99.44) | 99.85 (99.53) | * | 98.59 (95.29) | 99.90 (99.60) | 99.91 (99.59) |
| **RNS 05.1.2A** | 98.63 (93.32) | 98.63 (93.19) | 98.63 (93.32) | 98.63 (93.32) | 98.63 (93.21) | 98.63 (93.25) | 98.63 (93.32) | 98.63 (93.30) | 98.61 (93.27) | 98.63 (93.32) | 98.60 (92.56) | 98.61 (92.72) | 98.62 (92.74) | 98.64 (91.57) | 98.63 (93.02) | 98.63 (93.15) | 98.61 (93.27) | 98.55 (93.13) | 98.57 (93.16) | * | 98.66 (93.15) | 98.62 (93.31) |
| **RNS 07.7.3B** | 99.97 (99.70) | 99.96 (99.47) | 99.97 (99.70) | 99.97 (99.70) | 99.96 (99.45) | 99.97 (99.62) | 99.97 (99.70) | 99.96 (99.70) | 99.95 (99.63) | 99.96 (99.69) | 99.92 (98.95) | 99.94 (99.10) | 99.94 (98.94) | 99.94 (97.21) | 99.93 (99.43) | 99.94 (99.61) | 99.93 (99.53) | 99.88 (99.55) | 99.89 (99.60) | 98.68 (95.22) | * | 99.95 (99.68) |
| **RNS 08.23.3.1A** | 100.00 (99.52) | 99.99 (99.04) | 100.00 (99.52) | 100.00 (99.52) | 99.99 (99.04) | 100.00 (99.42) | 100.00 (99.52) | 100.00 (99.49) | 99.98 (99.45) | 100.00 (99.51) | 99.95 (98.77) | 99.98 (98.87) | 99.98 (98.16) | 99.98 (96.48) | 99.97 (98.82) | 99.98 (98.97) | 99.94 (99.31) | 99.91 (98.76) | 99.93 (98.85) | 98.68 (94.56) | 99.96 (98.93) | * |
