# Supplementary material for: Comparative genomics and pangenome-oriented studies reveal high homogeneity of the agronomically relevant enterobacterial plant pathogen Dickeya solani
Source: BMC Genomics. 2020 Jun 29;21:449. doi: 10.1186/s12864-020-06863-w (PMC7325237; doi:10.1186/s12864-020-06863-w)
Supplement: Supplementary file 2 — Additional file 2: Table S2. ANIm values calculated for the studied Dickeya solani genomes. Description of data: MUMmer calculation of ANI (ANIm) was performed with the use of JSpecies [106]. The upper number refers to ANIm value while the lower depicted in parentheses is the percentage of the aligned sequences. [file 12864_2020_6863_MOESM2_ESM.docx]

|  | **IFB0099** | **IFB0158** | **IFB0167** | **IFB0212** | **IFB0221** | **IFB0223** | **IFB0231** | **IFB0311** | **IFB0417** | **IFB0421** | **IFB0487** | **IFB0695** | **IPO 2222** | **GBBC 2040** | **MK 10** | **MK 16** | **D s0432-1** | **PPO 9019** | **PPO 9134** | **RNS 05.1.2A** | **RNS 07.7.3B** | **RNS 08.23.3.1A** |
| --- | --- | --- | --- | --- | --- | --- | --- | --- | --- | --- | --- | --- | --- | --- | --- | --- | --- | --- | --- | --- | --- | --- |
| **IFB0099** | * | 99.99 (99.93) | 99.99 (100.00) | 99.99 (100.00) | 99.99 (99.94) | 99.99 (99.94) | 99.99 (100.00) | 99.98 (100.00) | 99.98 (99.95) | 99.99 (100.00) | 99.98 (99.25) | 99.99 (99.39) | 99.99 (99.30) | 99.98 (98.79) | 99.99 (99.54) | 99.99 (99.49) | 99.99 (99.81) | 99.91 (99.35) | 99.92 (99.47) | 98.78 (95.92) | 99.96 (99.48) | 99.99 (99.97) |
| **IFB0158** | 99.99 (99.99) | * | 99.99 (99.99) | 99.99 (99.99) | 99.99 (99.99) | 99.99 (99.93) | 99.99 (99.99) | 99.99 (99.99) | 99.97 (99.94) | 99.98 (99.99) | 99.97 (99.24) | 99.98 (99.38) | 99.98 (99.65) | 99.98 (99.13) | 99.98 (99.81) | 99.99 (99.78) | 99.99 (99.82) | 99.90 (99.69) | 99.92 (99.75) | 98.79 (96.22) | 99.95 (99.78) | 99.98 (99.97) |
| **IFB0167** | 99.99 (100.00) | 99.99 (99.93) | * | 99.99 (100.00) | 99.99 (99.94) | 99.99 (99.94) | 99.99 (100.00) | 99.98 (100.00) | 99.98 (99.95) | 99.99 (100.00) | 99.98 (99.25) | 99.99 (99.39) | 99.99 (99.25) | 99.99 (98.74) | 99.99 (99.52) | 99.99 (99.47) | 99.99 (99.81) | 99.91 (99.31) | 99.92 (99.45) | 98.78 (95.87) | 99.96 (99.49) | 99.99 (99.98) |
| **IFB0212** | 99.99 (100.00) | 99.99 (99.94) | 99.99 (100.00) | * | 99.99 (99.95) | 99.99 (99.94) | 99.99 (100.00) | 99.98 (100.00) | 99.98 (99.95) | 99.99 (100.00) | 99.98 (99.25) | 99.99 (99.39) | 99.99 (99.38) | 99.99 (98.86) | 99.99 (99.62) | 99.99 (99.56) | 99.99 (99.82) | 99.91 (99.42) | 99.92 (99.55) | 98.78 (95.98) | 99.96 (99.56) | 99.99 (99.98) |
| **IFB0221** | 99.99 (99.99) | 99.99 (99.98) | 99.99 (99.99) | 99.99 (99.99) | * | 99.99 (99.93) | 99.99 (99.99) | 99.99 (99.99) | 99.97 (99.95) | 99.98 (99.99) | 99.97 (99.24) | 99.98 (99.38) | 99.98 (99.64) | 99.98 (99.12) | 99.98 (99.81) | 99.98 (99.80) | 99.98 (99.81) | 99.90 (99.69) | 99.92 (99.75) | 98.79 (96.21) | 99.95 (99.78) | 99.98 (99.97) |
| **IFB0223** | 99.99 (100.00) | 99.99 (99.93) | 99.99 (100.00) | 99.99 (100.00) | 99.99 (99.94) | * | 99.99 (100.00) | 99.98 (100.00) | 99.98 (99.95) | 99.99 (100.00) | 99.98 (99.25) | 99.99 (99.40) | 99.99 (99.30) | 99.98 (98.78) | 99.99 (99.54) | 99.99 (99.49) | 99.99 (99.81) | 99.91 (99.35) | 99.92 (99.46) | 98.78 (95.92) | 99.96 (99.48) | 99.99 (99.97) |
| **IFB0231** | 99.99 (100.00) | 99.99 (99.93) | 99.99 (100.00) | 99.99 (100.00) | 99.99 (99.94) | 99.99 (99.94) | * | 99.98 (100.00) | 99.98 (99.95) | 99.99 (100.00) | 99.98 (99.25) | 99.99 (99.39) | 99.99 (99.30) | 99.99 (98.78) | 99.99 (99.54) | 99.99 (99.48) | 99.99 (99.81) | 99.91 (99.34) | 99.92 (99.47) | 98.78 (95.91) | 99.96 (99.48) | 99.99 (99.97) |
| **IFB0311** | 99.98 (100.00) | 99.99 (99.94) | 99.98 (100.00) | 99.98 (100.00) | 99.99 (99.95) | 99.98 (99.94) | 99.98 (100.00) | * | 99.97 (99.95) | 99.98 (100.00) | 99.97 (99.25) | 99.98 (99.39) | 99.99 (99.34) | 99.98 (98.83) | 99.99 (99.58) | 99.99 (99.54) | 99.99 (99.82) | 99.91 (99.41) | 99.92 (99.53) | 98.78 (95.97) | 99.96 (99.54) | 99.99 (99.98) |
| **IFB0417** | 99.98 (100.00) | 99.97 (99.93) | 99.98 (100.00) | 99.98 (100.00) | 99.97 (99.94) | 99.98 (99.94) | 99.98 (100.00) | 99.97 (100.00) | * | 99.98 (100.00) | 99.97 (99.25) | 99.98 (99.39) | 99.98 (99.30) | 99.97 (98.79) | 99.98 (99.54) | 99.98 (99.49) | 99.97 (99.81) | 99.89 (99.35) | 99.91 (99.47) | 98.76 (95.92) | 99.95 (99.48) | 99.98 (99.98) |
| **IFB0421** | 99.99 (100.00) | 99.98 (99.93) | 99.99 (100.00) | 99.99 (100.00) | 99.98 (99.94) | 99.99 (99.94) | 99.99 (100.00) | 99.98 (100.00) | 99.98 (99.95) | * | 99.98 (99.25) | 99.99 (99.39) | 99.99 (99.30) | 99.98 (98.79) | 99.99 (99.55) | 99.99 (99.49) | 99.99 (99.81) | 99.90 (99.35) | 99.91 (99.48) | 98.78 (95.93) | 99.96 (99.49) | 99.99 (99.98) |
| **IFB0487** | 99.98 (100.00) | 99.97 (99.93) | 99.98 (100.00) | 99.98 (100.00) | 99.97 (99.94) | 99.98 (99.94) | 99.98 (100.00) | 99.97 (100.00) | 99.97 (99.95) | 99.98 (100.00) | * | 99.98 (99.39) | 99.97 (99.30) | 99.97 (98.78) | 99.97 (99.54) | 99.98 (99.49) | 99.97 (99.81) | 99.89 (99.35) | 99.91 (99.47) | 98.76 (95.89) | 99.94 (99.48) | 99.98 (99.98) |
| **IFB0695** | 99.99 (100.00) | 99.98 (99.92) | 99.99 (100.00) | 99.99 (100.00) | 99.98 (99.94) | 99.99 (99.94) | 99.99 (100.00) | 99.98 (100.00) | 99.98 (99.95) | 99.99 (100.00) | 99.98 (99.25) | * | 99.99 (99.30) | 99.98 (98.78) | 99.98 (99.54) | 99.99 (99.49) | 99.99 (99.82) | 99.90 (99.35) | 99.92 (99.47) | 98.77 (95.91) | 99.95 (99.49) | 99.98 (99.98) |
| **IPO 2222** | 99.99 (99.81) | 99.98 (99.79) | 99.99 (99.81) | 99.99 (99.81) | 99.98 (99.80) | 99.99 (99.74) | 99.99 (99.81) | 99.99 (99.81) | 99.98 (99.76) | 99.99 (99.81) | 99.98 (99.05) | 99.99 (99.20) | * | 99.99 (99.29) | 99.98 (99.79) | 99.99 (99.79) | 99.99 (99.65) | 99.90 (99.71) | 99.92 (99.71) | 98.79 (96.15) | 99.95 (99.72) | 99.98 (99.79) |
| **GBBC 2040** | 99.99 (99.44) | 99.98 (99.42) | 99.99 (99.44) | 99.99 (99.44) | 99.98 (99.43) | 99.99 (99.37) | 99.99 (99.44) | 99.99 (99.44) | 99.97 (99.39) | 99.98 (99.44) | 99.97 (98.69) | 99.98 (98.83) | 99.99 (99.44) | * | 99.98 (99.42) | 99.99 (99.42) | 99.98 (99.27) | 99.90 (99.34) | 99.91 (99.33) | 98.79 (96.07) | 99.95 (99.35) | 99.98 (99.42) |
| **MK 10** | 99.99 (99.92) | 99.98 (99.90) | 99.99 (99.92) | 99.99 (99.92) | 99.98 (99.91) | 99.99 (99.86) | 99.99 (99.92) | 99.99 (99.92) | 99.98 (99.88) | 99.99 (99.92) | 99.97 (99.18) | 99.98 (99.32) | 99.98 (99.72) | 99.98 (99.21) | * | 99.99 (99.86) | 99.99 (99.81) | 99.90 (99.74) | 99.91 (99.77) | 98.79 (96.05) | 99.96 (99.78) | 99.98 (99.91) |
| **MK 16** | 99.99 (99.96) | 99.98 (99.94) | 99.99 (99.96) | 99.99 (99.96) | 99.98 (99.95) | 99.99 (99.89) | 99.99 (99.96) | 99.99 (99.95) | 99.98 (99.91) | 99.99 (99.96) | 99.98 (99.20) | 99.99 (99.34) | 99.99 (99.74) | 99.98 (99.23) | 99.99 (99.87) | * | 99.99 (99.79) | 99.91 (99.76) | 99.90 (99.83) | 98.79 (96.20) | 99.96 (99.85) | 99.99 (99.94) |
| **D s0432-1** | 99.99 (100.00) | 99.99 (99.95) | 99.99 (100.00) | 99.99 (100.00) | 99.98 (99.96) | 99.99 (99.94) | 99.99 (100.00) | 99.99 (100.00) | 99.98 (99.95) | 99.99 (100.00) | 99.97 (99.25) | 99.99 (99.39) | 99.99 (99.34) | 99.98 (98.82) | 99.99 (99.56) | 99.99 (99.58) | * | 99.91 (99.39) | 99.91 (99.51) | 98.80 (95.97) | 99.96 (99.53) | 99.98 (99.99) |
| **PPO 9019** | 99.91 (99.99) | 99.90 (99.96) | 99.91 (99.99) | 99.91 (99.99) | 99.90 (99.97) | 99.91 (99.92) | 99.91 (99.99) | 99.91 (99.99) | 99.90 (99.94) | 99.91 (99.99) | 99.89 (99.23) | 99.91 (99.37) | 99.90 (99.80) | 99.90 (99.28) | 99.90 (99.90) | 99.91 (99.91) | 99.91 (99.82) | * | 99.85 (99.94) | 98.71 (96.37) | 99.88 (99.97) | 99.90 (99.97) |
| **PPO 9134** | 99.92 (99.97) | 99.92 (99.94) | 99.92 (99.97) | 99.92 (99.97) | 99.92 (99.95) | 99.92 (99.91) | 99.92 (99.97) | 99.92 (99.97) | 99.91 (99.92) | 99.92 (99.97) | 99.91 (99.21) | 99.92 (99.36) | 99.92 (99.72) | 99.91 (99.20) | 99.92 (99.85) | 99.92 (99.87) | 99.92 (99.80) | 99.85 (99.87) | * | 98.72 (96.34) | 99.90 (99.94) | 99.92 (99.96) |
| **RNS 05.1.2A** | 98.78 (94.00) | 98.79 (93.98) | 98.78 (94.00) | 98.78 (94.00) | 98.79 (93.99) | 98.78 (93.94) | 98.78 (94.00) | 98.78 (94.00) | 98.77 (93.96) | 98.78 (94.00) | 98.77 (93.26) | 98.78 (93.40) | 98.80 (93.79) | 98.80 (93.57) | 98.79 (93.92) | 98.79 (93.90) | 98.80 (93.93) | 98.71 (93.89) | 98.73 (93.95) | * | 98.82 (93.90) | 98.78 (94.00) |
| **RNS 07.7.3B** | 99.96 (99.99) | 99.95 (99.96) | 99.96 (99.99) | 99.96 (99.99) | 99.95 (99.97) | 99.96 (99.93) | 99.96 (99.99) | 99.96 (99.99) | 99.95 (99.95) | 99.96 (99.99) | 99.94 (99.24) | 99.96 (99.38) | 99.96 (99.71) | 99.95 (99.19) | 99.96 (99.86) | 99.96 (99.88) | 99.96 (99.83) | 99.88 (99.87) | 99.90 (99.92) | 98.82 (96.28) | * | 99.96 (99.98) |
| **RNS 08.23.3.1A** | 99.99 (99.75) | 99.98 (99.68) | 99.99 (99.75) | 99.99 (99.75) | 99.98 (99.70) | 99.99 (99.69) | 99.99 (99.75) | 99.99 (99.75) | 99.98 (99.71) | 99.99 (99.75) | 99.98 (99.01) | 99.98 (99.15) | 99.98 (99.10) | 99.98 (98.59) | 99.98 (99.35) | 99.99 (99.34) | 99.98 (99.55) | 99.90 (99.16) | 99.92 (99.28) | 98.78 (95.72) | 99.96 (99.30) | * |
