# Supplementary material for: Comparative genomics and pangenome-oriented studies reveal high homogeneity of the agronomically relevant enterobacterial plant pathogen Dickeya solani
Source: BMC Genomics. 2020 Jun 29;21:449. doi: 10.1186/s12864-020-06863-w (PMC7325237; doi:10.1186/s12864-020-06863-w)
Supplement: Supplementary file 3 — Additional file 3: Table S3. Correlation indexes of the tetra-nucleotide signatures computed for the studied Dickeya solani genomes. Description of data: Computation of the correlation indexes of the tetra-nucleotide signatures was conducted with JSpecies [106]. [file 12864_2020_6863_MOESM3_ESM.docx]

|  | **IFB0099** | **IFB0158** | **IFB0167** | **IFB0212** | **IFB0221** | **IFB0223** | **IFB0231** | **IFB0311** | **IFB0417** | **IFB0421** | **IFB0487** | **IFB0695** | **IPO 2222** | **GBBC 2040** | **MK 10** | **MK 16** | **D s0432-1** | **PPO 9019** | **PPO 9134** | **RNS 05.1.2A** | **RNS 07.7.3B** | **RNS 08.23.3.1A** |
| --- | --- | --- | --- | --- | --- | --- | --- | --- | --- | --- | --- | --- | --- | --- | --- | --- | --- | --- | --- | --- | --- | --- |
| **IFB0099** | * | 0.99996 | 1.0 | 0.99999 | 0.99996 | 1.0 | 1.0 | 1.0 | 0.99994 | 1.0 | 0.99994 | 0.99999 | 0.99994 | 0.99989 | 0.99994 | 0.99995 | 0.99997 | 0.99995 | 0.99995 | 0.99981 | 0.99995 | 0.99999 |
| **IFB0158** | 0.99996 | * | 0.99997 | 0.99998 | 1.0 | 0.99996 | 0.99997 | 0.99998 | 0.99994 | 0.99997 | 0.99994 | 0.99996 | 0.99999 | 0.99993 | 0.99998 | 0.99999 | 0.99996 | 0.99999 | 0.99999 | 0.99985 | 1.0 | 0.99998 |
| **IFB0167** | 1.0 | 0.99997 | * | 1.0 | 0.99997 | 1.0 | 1.0 | 1.0 | 0.99994 | 1.0 | 0.99994 | 0.99999 | 0.99995 | 0.99989 | 0.99994 | 0.99995 | 0.99998 | 0.99995 | 0.99995 | 0.99981 | 0.99996 | 0.99999 |
| **IFB0212** | 0.99999 | 0.99998 | 1.0 | * | 0.99998 | 0.99999 | 1.0 | 1.0 | 0.99995 | 1.0 | 0.99995 | 0.99999 | 0.99996 | 0.99991 | 0.99996 | 0.99997 | 0.99997 | 0.99997 | 0.99997 | 0.99983 | 0.99998 | 1.0 |
| **IFB0221** | 0.99996 | 1.0 | 0.99997 | 0.99998 | * | 0.99996 | 0.99997 | 0.99998 | 0.99994 | 0.99997 | 0.99994 | 0.99996 | 0.99999 | 0.99993 | 0.99998 | 0.99999 | 0.99996 | 0.99999 | 0.99999 | 0.99985 | 1.0 | 0.99998 |
| **IFB0223** | 1.0 | 0.99996 | 1.0 | 0.99999 | 0.99996 | * | 1.0 | 1.0 | 0.99994 | 1.0 | 0.99994 | 0.99999 | 0.99994 | 0.99989 | 0.99994 | 0.99994 | 0.99997 | 0.99995 | 0.99995 | 0.99981 | 0.99995 | 0.99999 |
| **IFB0231** | 1.0 | 0.99997 | 1.0 | 1.0 | 0.99997 | 1.0 | * | 1.0 | 0.99994 | 1.0 | 0.99994 | 0.99999 | 0.99995 | 0.99989 | 0.99994 | 0.99995 | 0.99998 | 0.99995 | 0.99995 | 0.99982 | 0.99996 | 0.99999 |
| **IFB0311** | 1.0 | 0.99998 | 1.0 | 1.0 | 0.99998 | 1.0 | 1.0 | * | 0.99995 | 1.0 | 0.99995 | 0.99999 | 0.99996 | 0.9999 | 0.99995 | 0.99996 | 0.99998 | 0.99996 | 0.99996 | 0.99983 | 0.99997 | 1.0 |
| **IFB0417** | 0.99994 | 0.99994 | 0.99994 | 0.99995 | 0.99994 | 0.99994 | 0.99994 | 0.99995 | * | 0.99995 | 1.0 | 0.99995 | 0.99993 | 0.99988 | 0.99992 | 0.99993 | 0.99994 | 0.99993 | 0.99993 | 0.99976 | 0.99993 | 0.99995 |
| **IFB0421** | 1.0 | 0.99997 | 1.0 | 1.0 | 0.99997 | 1.0 | 1.0 | 1.0 | 0.99995 | * | 0.99995 | 0.99999 | 0.99995 | 0.9999 | 0.99995 | 0.99995 | 0.99998 | 0.99996 | 0.99996 | 0.99982 | 0.99996 | 0.99999 |
| **IFB0487** | 0.99994 | 0.99994 | 0.99994 | 0.99995 | 0.99994 | 0.99994 | 0.99994 | 0.99995 | 1.0 | 0.99995 | * | 0.99995 | 0.99993 | 0.99988 | 0.99992 | 0.99993 | 0.99993 | 0.99993 | 0.99993 | 0.99976 | 0.99993 | 0.99995 |
| **IFB0695** | 0.99999 | 0.99996 | 0.99999 | 0.99999 | 0.99996 | 0.99999 | 0.99999 | 0.99999 | 0.99995 | 0.99999 | 0.99995 | * | 0.99994 | 0.9999 | 0.99993 | 0.99994 | 0.99997 | 0.99995 | 0.99995 | 0.9998 | 0.99995 | 0.99999 |
| **IPO 2222** | 0.99994 | 0.99999 | 0.99995 | 0.99996 | 0.99999 | 0.99994 | 0.99995 | 0.99996 | 0.99993 | 0.99995 | 0.99993 | 0.99994 | * | 0.99996 | 0.99999 | 0.99999 | 0.99996 | 0.99999 | 0.99999 | 0.99985 | 0.99999 | 0.99996 |
| **GBBC 2040** | 0.99989 | 0.99993 | 0.99989 | 0.99991 | 0.99993 | 0.99989 | 0.99989 | 0.9999 | 0.99988 | 0.9999 | 0.99988 | 0.9999 | 0.99996 | * | 0.99991 | 0.99993 | 0.9999 | 0.99994 | 0.99993 | 0.99979 | 0.99993 | 0.9999 |
| **MK 10** | 0.99994 | 0.99998 | 0.99994 | 0.99996 | 0.99998 | 0.99994 | 0.99994 | 0.99995 | 0.99992 | 0.99995 | 0.99992 | 0.99993 | 0.99999 | 0.99991 | * | 0.99999 | 0.99996 | 0.99998 | 0.99999 | 0.99986 | 0.99999 | 0.99996 |
| **MK 16** | 0.99995 | 0.99999 | 0.99995 | 0.99997 | 0.99999 | 0.99994 | 0.99995 | 0.99996 | 0.99993 | 0.99995 | 0.99993 | 0.99994 | 0.99999 | 0.99993 | 0.99999 | * | 0.99996 | 0.99999 | 0.99999 | 0.99987 | 1.0 | 0.99997 |
| **D s0432-1** | 0.99997 | 0.99996 | 0.99998 | 0.99997 | 0.99996 | 0.99997 | 0.99998 | 0.99998 | 0.99994 | 0.99998 | 0.99993 | 0.99997 | 0.99996 | 0.9999 | 0.99996 | 0.99996 | * | 0.99994 | 0.99995 | 0.99983 | 0.99995 | 0.99998 |
| **PPO 9019** | 0.99995 | 0.99999 | 0.99995 | 0.99997 | 0.99999 | 0.99995 | 0.99995 | 0.99996 | 0.99993 | 0.99996 | 0.99993 | 0.99995 | 0.99999 | 0.99994 | 0.99998 | 0.99999 | 0.99994 | * | 0.99999 | 0.99986 | 0.99999 | 0.99997 |
| **PPO 9134** | 0.99995 | 0.99999 | 0.99995 | 0.99997 | 0.99999 | 0.99995 | 0.99995 | 0.99996 | 0.99993 | 0.99996 | 0.99993 | 0.99995 | 0.99999 | 0.99993 | 0.99999 | 0.99999 | 0.99995 | 0.99999 | * | 0.99986 | 1.0 | 0.99997 |
| **RNS 05.1.2A** | 0.99981 | 0.99985 | 0.99981 | 0.99983 | 0.99985 | 0.99981 | 0.99982 | 0.99983 | 0.99976 | 0.99982 | 0.99976 | 0.9998 | 0.99985 | 0.99979 | 0.99986 | 0.99987 | 0.99983 | 0.99986 | 0.99986 | * | 0.99986 | 0.99983 |
| **RNS 07.7.3B** | 0.99995 | 1.0 | 0.99996 | 0.99998 | 1.0 | 0.99995 | 0.99996 | 0.99997 | 0.99993 | 0.99996 | 0.99993 | 0.99995 | 0.99999 | 0.99993 | 0.99999 | 1.0 | 0.99995 | 0.99999 | 1.0 | 0.99986 | * | 0.99997 |
| **RNS 08.23.3.1A** | 0.99999 | 0.99998 | 0.99999 | 1.0 | 0.99998 | 0.99999 | 0.99999 | 1.0 | 0.99995 | 0.99999 | 0.99995 | 0.99999 | 0.99996 | 0.9999 | 0.99996 | 0.99997 | 0.99998 | 0.99997 | 0.99997 | 0.99983 | 0.99997 | * |
