# Supplementary material for: Comparative genomics and pangenome-oriented studies reveal high homogeneity of the agronomically relevant enterobacterial plant pathogen Dickeya solani
Source: BMC Genomics. 2020 Jun 29;21:449. doi: 10.1186/s12864-020-06863-w (PMC7325237; doi:10.1186/s12864-020-06863-w)
Supplement: Supplementary file 4 — Additional file 4: Table S4. The functions of the unique Dickeya solani COGs. Description of data: a The first category was attributed by the BPGA v. 1.3 software. If it differed from the category currently allocated to certain COG IDs in the COG database, the up-to-date assignment after the slash mark is depicted. COG categories: A - RNA processing and modification, B - chromatin structure and dynamics, C - energy production and conversion, D - cell cycle control, cell division, chromosome partitioning, E - amino acid transport and metabolism, F - nucleotide transport and metabolism, G - carbohydrate transport and metabolism, H - coenzyme transport and metabolism, I - lipid transport and metabolism, J - translation, ribosomal structure and biogenesis, K - transcription, L - replication, recombination and repair, M - cell wall/membrane/envelope biogenesis, N - cell motility, O - post-translational modification, protein turnover and chaperones, P - inorganic ion transport and metabolism, Q - secondary metabolites biosynthesis, transport and catabolism, R - general function prediction only, S - function unknown, T - signal transduction mechanisms, U - intracellular trafficking, secretion and vesicular transport, V - defence mechanisms, W - extracellular structures, X - mobilome: prophages, transposons, Y - nuclear structure and Z – cytoskeleton. [file 12864_2020_6863_MOESM4_ESM.docx]

| **Genome** | **COG category^a^** | **COG ID** | **Annotation** | **Present in *D. dadantii* 3937** |
| --- | --- | --- | --- | --- |
| IFB0417 | L\LX | COG0582 | Integrase | + |
| IFB0417 | Q | COG1020 | Non-ribosomal peptide synthetase component F | + |
| IFB0417 | C\I | COG1804 | Crotonobetainyl-CoA:carnitine CoA-transferase CaiB and related acyl-CoA transferases | + |
| IFB0417 | R\X | COG5301 | Phage-related tail fibre protein | + |
| IFB0421 | G | COG2133 | Glucose/arabinose dehydrogenase, beta-propeller fold | + |
| IFB0487 | I | COG0439 | Biotin carboxylase | + |
| IFB0487 | K | COG0583 | DNA-binding transcriptional regulator, LysR family | + |
| IFB0487 | E | COG0765 | ABC-type amino acid transport system, permease component | + |
| IFB0487 | NT | COG0840 | Methyl-accepting chemotaxis protein | + |
| IFB0487 | NT | COG0840 | Methyl-accepting chemotaxis protein | + |
| IFB0487 | I | COG1024 | Enoyl-CoA hydratase/carnithine racemase | + |
| IFB0487 | E | COG2049 | Allophanate hydrolase subunit 1 | + |
| IFB0487 | Q | COG3321 | Acyl transferase domain in polyketide synthase (PKS) enzymes | - |
| MK10 | K | COG1737 | DNA-binding transcriptional regulator, MurR/RpiR family, contains HTH and SIS domains | + |
| RNS 05.1.2A | U | COG3210 | Large exoprotein involved in heme utilization or adhesion | + |
| RNS 05.1.2A | S | COG4458 | Uncharacterized protein | + |
| RNS 05.1.2A | KL | COG1061 | Superfamily II DNA or RNA helicase | + |
| RNS 05.1.2A | P | COG1055 | Na+/H+ antiporter NhaD or related arsenite permease | - |
| RNS 05.1.2A | R\x | COG4626 | Phage terminase-like protein, large subunit, contains N-terminal HTH domain | - |
| RNS 05.1.2A | S\K | COG3177 | Fic family protein | + |
| RNS 05.1.2A | S\X | COG5283 | Phage-related tail protein | + |
| RNS 05.1.2A | L\X | COG0582 | Integrase | + |
| RNS 05.1.2A | R\T | COG1073 | Fermentation-respiration switch protein FrsA, has esterase activity, DUF1100 family | + |
| RNS 05.1.2A | M | COG2148 | Sugar transferase involved in LPS biosynthesis (colanic, teichoic acid) | + |
| RNS 05.1.2A | G | COG2814 | Predicted arabinose efflux permease, MFS family | + |
| RNS 05.1.2A | F | COG2131 | Deoxycytidylate deaminase | - |
| RNS 05.1.2A | S\X | COG4695 | Phage portal protein BeeE | + |
| RNS 05.1.2A | R\X | COG4653 | Predicted phage phi-C31 gp36 major capsid-like protein | - |
| RNS 05.1.2A | GEPR | COG0477 | MFS family permease | + |
| RNS 05.1.2A | S | COG5484 | Uncharacterized protein YjcR | - |
| RNS 05.1.2A | S\O | COG1262 | Formylglycine-generating enzyme, required for sulfatase activity, contains SUMF1/FGE domain | + |
| RNS 05.1.2A | R\L | COG1201 | Lhr-like helicase | - |
| RNS 05.1.2A | R\X | COG5518 | Bacteriophage capsid portal protein | - |
| RNS 05.1.2A | S\X | COG4643 | Uncharacterized domain associated with phage/plasmid primase | + |
| RNS 05.1.2A | K | COG0583 | DNA-binding transcriptional regulator, LysR family | + |
| RNS 05.1.2A | S\X | COG5283 | Phage-related tail protein | + |
| RNS 05.1.2A | R | COG3500 | Phage protein D | + |
| RNS 05.1.2A | L\LX | COG0582 | Integrase | + |
| RNS 05.1.2A | R\X | COG4386 | Mu-like prophage tail sheath protein gpL | - |
| RNS 05.1.2A | V | COG0577 | ABC-type antimicrobial peptide transport system, permease component | + |
| RNS 05.1.2A | S\X | COG3299 | Uncharacterized phage protein gp47/JayE | + |
| RNS 05.1.2A | R\X | COG1783 | Phage terminase large subunit | - |
| RNS 05.1.2A | J | COG0154 | Asp-tRNAAsn/Glu-tRNAGln amidotransferase A subunit or related amidase | + |
| RNS 05.1.2A | V | COG4268 | 5-methylcytosine-specific restriction endonuclease McrBC, regulatory subunit McrC | - |
| RNS 05.1.2A | C\S | COG4313 | Uncharacterized conserved protein | + |
| RNS 05.1.2A | S\X | COG3778 | Uncharacterized protein YmfQ in lambdoid prophage, DUF2313 family | - |
| RNS 05.1.2A | H | COG1541 | Phenylacetate-coenzyme A ligase PaaK, adenylate-forming domain family | + |
| RNS 05.1.2A | R\X | COG3740 | Phage head maturation protease | - |
| RNS 05.1.2A | L | COG0338 | Site-specific DNA-adenine methylase | + |
| RNS 05.1.2A | V | COG1401 | 5-methylcytosine-specific restriction endonuclease McrBC, GTP-binding regulatory subunit McrB | - |
| RNS 05.1.2A | EH | COG0115 | Branched-chain amino acid aminotransferase/4-amino-4-deoxychorismate lyase | + |
| RNS 05.1.2A | R\X | COG5301 | Phage-related tail fibre protein | + |
| RNS 05.1.2A | L\LH | COG0582 | Integrase | + |
| RNS 05.1.2A | R | COG3179 | Predicted chitinase | + |
| RNS 05.1.2A | K | COG0583 | DNA-binding transcriptional regulator, LysR family | + |
| RNS 05.1.2A | V\M | COG1136 | ABC-type lipoprotein export system, ATPase component | + |
| RNS 05.1.2A | V | COG0732 | Restriction endonuclease S subunit | - |
| RNS 05.1.2A | R | COG2085 | Predicted dinucleotide-binding enzyme | + |
| RNS 05.1.2A | GEPR | COG0477 | MFS family permease | + |
| RNS 05.1.2A | S\R | COG4226 | Predicted nuclease of the RNAse H fold, HicB family | + |
| RNS 05.1.2A | R\X | COG5301 | Phage-related tail fibre protein | + |
| RNS 05.1.2A | S | COG3567 | Uncharacterized protein | - |
| RNS 05.1.2A | G | COG4193 | Beta-N-acetylglucosaminidase | - |
| RNS 05.1.2A | S\X | COG5283 | Phage-related tail protein | + |
| RNS 05.1.2A | K | COG1396 | Transcriptional regulator, contains XRE-family HTH domain | + |
| RNS 05.1.2A | K | COG2207 | AraC-type DNA-binding domain and AraC-containing proteins | + |
| RNS 05.1.2A | S\X | COG3299 | Uncharacterized phage protein gp47/JayE | + |
| RNS 05.1.2A | R\X | COG4379 | Mu-like prophage tail protein gpP | - |
| RNS 05.1.2A | K | COG0640 | DNA-binding transcriptional regulator, ArsR family | + |
| RNS 05.1.2A | L | COG0507 | ATP-dependent exoDNAse (exonuclease V), alpha subunit, helicase superfamily I | + |
| RNS 05.1.2A | E | COG0436 | Aspartate/methionine/tyrosine aminotransferase | + |
| RNS 05.1.2A | S | COG4453 | Uncharacterized conserved protein, DUF1778 family | + |
| RNS 05.1.2A | K | COG1846 | DNA-binding transcriptional regulator, MarR family | + |
| RNS 05.1.2A | R\L | COG0433 | Archaeal DNA helicase HerA or a related bacterial ATPase, contains HAS-barrel and ATPase domains | - |
| RNS 05.1.2A | E | COG2423 | Ornithine cyclodeaminase/archaeal alanine dehydrogenase, mu-crystallin family | + |
| RNS 05.1.2A | R\X | COG3499 | Phage protein U | + |
| RNS 05.1.2A | K\X | COG2932 | Phage repressor protein C, contains Cro/C1-type HTH and peptisase s24 domains | + |
| RNS 05.1.2A | K | COG2207 | AraC-type DNA-binding domain and AraC-containing proteins | + |
| RNS 05.1.2A | JD\V | COG2026 | mRNA-degrading endonuclease RelE, toxin component of the RelBE toxin-antitoxin system | - |
| RNS 05.1.2A | K | COG2865 | Predicted transcriptional regulator, contains HTH domain | - |
| RNS 05.1.2A | R\C | COG0431 | AD(P)H-dependent FMN reductase | + |
| RNS 05.1.2A | S\P | COG2128 | Alkylhydroperoxidase family enzyme, contains CxxC motif | + |
| RNS 05.1.2A | E | COG1280 | Threonine/homoserine/homoserine lactone efflux protein | + |
| RNS 05.1.2A | S | COG5532 | Uncharacterized conserved protein YfdQ, DUF2303 family | - |
| RNS 05.1.2A | K\X | COG2932 | Phage repressor protein C, contains Cro/C1-type HTH and peptisase s24 domains | + |
| RNS 05.1.2A | R\W | COG3628 | Phage baseplate assembly protein W | + |
| RNS 05.1.2A | K | COG1396 | Transcriptional regulator, contains XRE-family HTH domain | + |
| RNS 05.1.2A | R\X | COG4228 | Mu-like prophage DNA circulation protein | - |
| RNS 05.1.2A | KT | COG1974 | SOS-response transcriptional repressor LexA (RecA-mediated autopeptidase) | + |
| RNS 05.1.2A | C | COG0778 | Nitroreductase | + |
| RNS 05.1.2A | S | COG4925 | Uncharacterized protein | - |
| RNS 05.1.2A | I\M | COG1835 | Peptidoglycan/LPS O-acetylase OafA/YrhL, contains acyltransferase and SGNH-hydrolase domains | + |
| RNS 05.1.2A | K\KX | COG3311 | Predicted DNA-binding transcriptional regulator AlpA | + |
| RNS 05.1.2A | R\X | COG5525 | Phage terminase, large subunit GpA | - |
| RNS 05.1.2A | S\X | COG4384 | Mu-like prophage protein gp45 | - |
| RNS 05.1.2A | L | COG4570 | Holliday junction resolvase RusA (prophage-encoded endonuclease) | - |
| RNS 05.1.2A | R\X | COG5004 | P2-like prophage tail protein X | + |
| RNS 05.1.2A | S | COG4381 | Mu-like prophage protein gp46 | - |
| RNS 05.1.2A | K | COG1309 | DNA-binding transcriptional regulator, AcrR family | + |
| RNS 05.1.2A | JD\V | COG2026 | mRNA-degrading endonuclease RelE, toxin component of the RelBE toxin-antitoxin system | - |
| RNS 05.1.2A | T\J | COG1734 | RNA polymerase-binding transcription factor DksA | + |
| RNS 05.1.2A | L | COG0338 | Site-specific DNA-adenine methylase | + |
| RNS 05.1.2A | L\X | COG2801 | Transposase InsO and inactivated derivatives | + |
| RNS 05.1.2A | G | COG1440 | Phosphotransferase system cellobiose-specific component IIB | + |
| RNS 05.1.2A | R\T | COG3550 | Serine/threonine protein kinase HipA, toxin component of the HipAB toxin-antitoxin module | + |
| RNS 05.1.2A | EH | COG0175 | 3'-phosphoadenosine 5'-phosphosulfate sulfotransferase (PAPS reductase)/FAD synthetase or related enzyme | + |
| RNS 05.1.2A | K | COG1396 | Transcriptional regulator, contains XRE-family HTH domain | + |
| RNS 05.1.2A | L\X | COG3747 | Phage terminase, small subunit | - |
| RNS 05.1.2A | L | COG0863 | DNA modification methylase | - |
| RNS 05.1.2A | TK\J | COG4725 | N6-adenosine-specific RNA methylase IME4 | - |
| RNS 05.1.2A | R\X | COG5614 | Bacteriophage head-tail adaptor | - |
| RNS 05.1.2A | U | COG3846 | Type IV secretory pathway, TrbL components | + |
| RNS 05.1.2A | L | COG0419 | DNA repair exonuclease SbcCD ATPase subunit | + |
| RNS 05.1.2A | L\X | COG2801 | Transposase InsO and inactivated derivatives | + |
| RNS 05.1.2A | S\FT | COG2357 | ppGpp synthetase catalytic domain (RelA/SpoT-type nucleotidyltranferase) | + |
| RNS 05.1.2A | L\LX | COG0582 | Integrase | + |
| RNS 05.1.2A | R\T | COG1073 | Fermentation-respiration switch protein FrsA, has esterase activity, DUF1100 family | + |
| RNS 05.1.2A | R\X | COG3499 | Phage protein U | + |
| RNS 05.1.2A | K | COG1396 | Transcriptional regulator, contains XRE-family HTH domain | + |
| RNS 05.1.2A | M | COG2951 | Membrane-bound lytic murein transglycosylase B | + |
